# Synthesis of pyrimidines from dinitrogen and carbon

**DOI:** 10.1093/nsr/nwac168

**Published:** 2022-08-24

**Authors:** Xianghui Shi, Qianru Wang, Chao Qin, Li-Jun Wu, Yuanjin Chen, Gao-Xiang Wang, Yongli Cai, Wenbo Gao, Teng He, Junnian Wei, Jianping Guo, Ping Chen, Zhenfeng Xi

**Affiliations:** Beijing National Laboratory for Molecular Sciences (BNLMS), Key Laboratory of Bioorganic Chemistry and Molecular Engineering of Ministry of Education, College of Chemistry, Peking University, Beijing 100871, China; Dalian Institute of Chemical Physics, Chinese Academy of Sciences, Dalian 116023, China; Dalian Institute of Chemical Physics, Chinese Academy of Sciences, Dalian 116023, China; Beijing National Laboratory for Molecular Sciences (BNLMS), Key Laboratory of Bioorganic Chemistry and Molecular Engineering of Ministry of Education, College of Chemistry, Peking University, Beijing 100871, China; Beijing National Laboratory for Molecular Sciences (BNLMS), Key Laboratory of Bioorganic Chemistry and Molecular Engineering of Ministry of Education, College of Chemistry, Peking University, Beijing 100871, China; Beijing National Laboratory for Molecular Sciences (BNLMS), Key Laboratory of Bioorganic Chemistry and Molecular Engineering of Ministry of Education, College of Chemistry, Peking University, Beijing 100871, China; Dalian Institute of Chemical Physics, Chinese Academy of Sciences, Dalian 116023, China; University of Chinese Academy of Sciences, Beijing 100049, China; Dalian Institute of Chemical Physics, Chinese Academy of Sciences, Dalian 116023, China; University of Chinese Academy of Sciences, Beijing 100049, China; Dalian Institute of Chemical Physics, Chinese Academy of Sciences, Dalian 116023, China; University of Chinese Academy of Sciences, Beijing 100049, China; Beijing National Laboratory for Molecular Sciences (BNLMS), Key Laboratory of Bioorganic Chemistry and Molecular Engineering of Ministry of Education, College of Chemistry, Peking University, Beijing 100871, China; Dalian Institute of Chemical Physics, Chinese Academy of Sciences, Dalian 116023, China; University of Chinese Academy of Sciences, Beijing 100049, China; Dalian Institute of Chemical Physics, Chinese Academy of Sciences, Dalian 116023, China; University of Chinese Academy of Sciences, Beijing 100049, China; State Key Laboratory of Catalysis, Dalian Institute of Chemical Physics, Chinese Academy of Sciences, Dalian 116023, China; Beijing National Laboratory for Molecular Sciences (BNLMS), Key Laboratory of Bioorganic Chemistry and Molecular Engineering of Ministry of Education, College of Chemistry, Peking University, Beijing 100871, China

**Keywords:** dinitrogen transformation, nitrogen fixation, N−C bond formation, homogeneous–heterogeneous synergy strategy, 15n-labeled pyrimidines

## Abstract

The element nitrogen and nitrogenous compounds are vital to life. The synthesis of nitrogen-containing compounds using dinitrogen as the nitrogen source, not through ammonia, is of great interest and great value but remains a grand challenge. Herein, we describe a strategy to realize this transformation by combining the heterogeneous approach with the homogeneous methodology. The N_2_ molecule was first fixed with carbon and LiH through a one-pot heterogeneous process, forming Li_2_CN_2_ as an ‘activated’ nitrogen source with high efficiency. Then subsequent homogeneous treatments of Li_2_CN_2_ to construct the organic synthon carbodiimide and the RNA/DNA building block pyrimidines were fulfilled. By using ^15^N_2_ as the feedstock, their corresponding ^15^N-labeled carbodiimide and pyrimidines were readily obtained. This homogeneous–heterogeneous synergy strategy will open a new chapter for N_2_ transformation.

## Teaser

As a new paradigm synergizing the heterogeneous approach with the homogeneous methodology for high-efficient direct dinitrogen utilization not via ammonia, N_2_ molecule, carbon, and LiH are combined to form Li_2_CN_2_, through which cytosine and thymine are synthesized.

## INTRODUCTION

**Figure 1.**
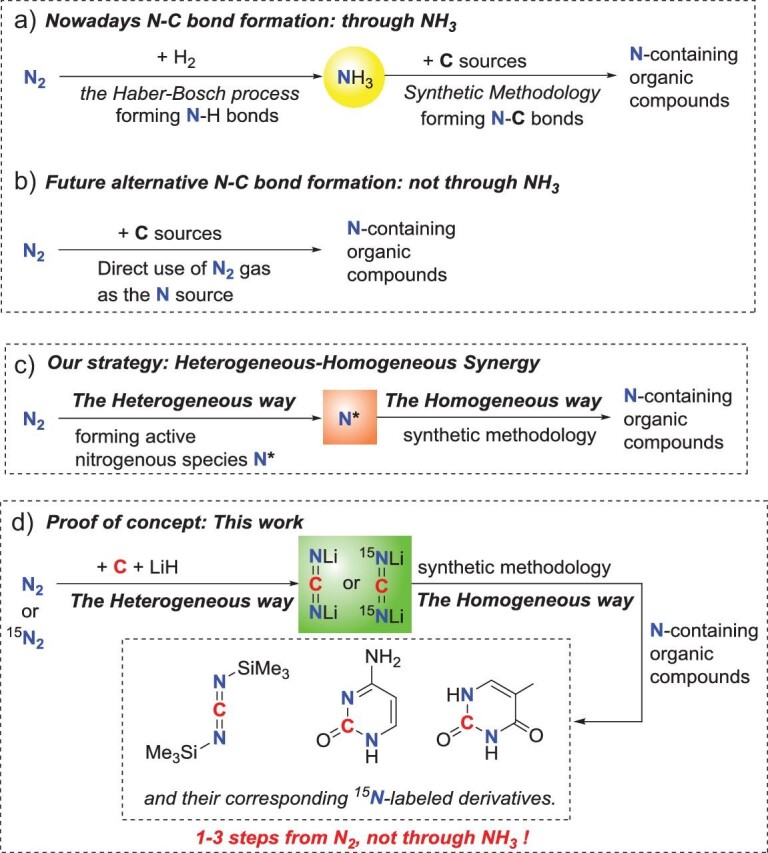
Manners of dinitrogen activation and transformation. (a) Current production and application of NH_3_. Almost all synthetic nitrogenous organic compounds are synthesized from NH_3_. (b) Synthesis of N-containing organic compounds directly from N_2_ molecule and carbon sources. (c) Our general strategy to achieve the above goal: taking advantage of the heterogeneous process to generate active intermediate nitrogenous species, followed by taking advantage of the homogeneous synthetic methodology to make value-added organic compounds. (d) This work is a proof of concept. Through a heterogeneous process, the N_2_ gas was first fixed with carbon and LiH to form reactive Li_2_CN_2_, which was then transformed into nitrogen-containing organic compounds through the homogeneous synthetic methodology.

The activation and transformation of dinitrogen (N_2_) gas are among the most intriguing challenges of modern chemistry. Chemists have explored this field with great effort from different perspectives. Still, until today, the Haber–Bosch process developed at the beginning of the twentieth century remains the dominant way to provide ‘activated’ nitrogen sources for human society (Fig. 1a) [1,2]. Nowadays, almost all synthetic nitrogenous compounds are synthesized from NH_3_. In other words, virtually all the N atoms in artificial N-containing compounds in human society are from the nitrogen in NH_3_. However, the current Haber–Bosch process and the applied routes from NH_3_ to various desired N-containing products are still far from satisfactory in many aspects [3–6].

An attractive alternative is to construct C–N bonds directly from N_2_ and carbon without using NH_3_ as the intermediate (Fig. 1b) [7–12]. Although this route can be considered an ideal direct use of N_2_ besides the Haber–Bosch process and several notable examples have been reported [13–22], this field is still in its infancy. The biggest problem holding back the field is finding out effective strategies that can ingeniously combine the activation of dinitrogen, a notoriously stable molecule, with the formation of nitrogen–carbon bonds.

Looking back through the literature, the most potent means of dinitrogen fixation are based on heterogeneous chemical reactions. In the meantime, the most effective ways to construct various C–N bonds are the homogeneous synthetic methodology. Thus, we envisage that combining a heterogeneous approach with a homogeneous synthetic methodology may be an efficient strategy to achieve the above goal (Fig. 1b). That is, as a concept (Fig. 1c), the N_2_ molecule is first turned into an active-enough nitrogenous species (N*) through a heterogeneous process and then a subsequent homogeneous reaction is performed to construct complex nitrogen-containing organic compounds, taking advantage of both research methodologies. As a proof of concept (Fig. 1d), in this work, the N_2_ gas was first fixed with carbon (expanded graphite) and LiH through a heterogeneous process, forming the vital reactive intermediate Li_2_CN_2_ with high selectivity and efficiency. Then subsequent homogeneous reactions of Li_2_CN_2_ were carried out to efficiently construct value-added nitrogen-containing organic compounds, including the organic synthon carbodiimide and pyrimidines. As the immediate building blocks for the synthesis of RNA/DNA, pyrimidines are indispensable raw materials in biomedical research, as well as essential elements in the preparation of biological drugs [23–25]. This cross-disciplinary strategy greatly simplifies the synthetic steps and reduces the production cost. Also, notably, by using the ^15^N_2_ gas as the feedstock, their corresponding ^15^N-labeled carbodiimide and pyrimidines could be readily obtained.

## RESULTS AND DISCUSSION

### Synthesis of Li_2_CN_2_ from LiH, graphite and N_2_ gas

**Figure 2.**
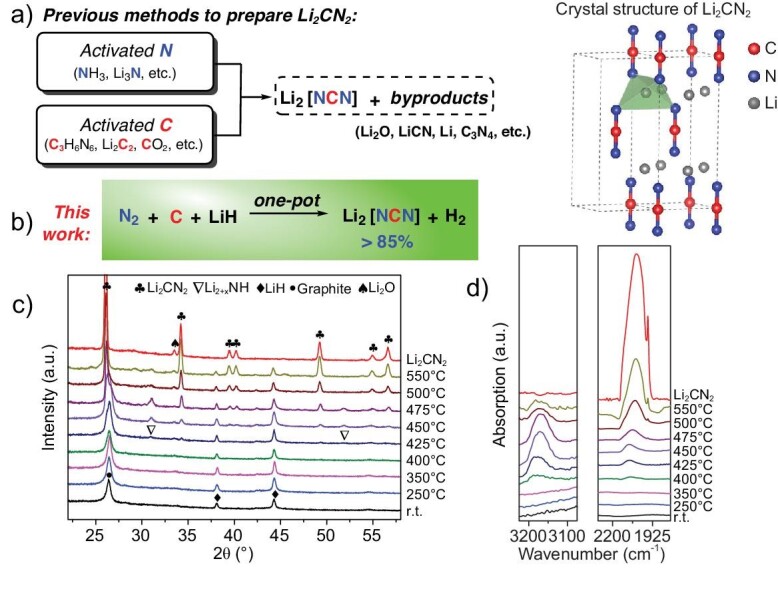
(a) Previous synthetic methods to prepare Li_2_CN_2_. (b) Synthesis of Li_2_CN_2_ from LiH, C and N_2_ via a one-pot procedure. The crystal structure of Li_2_CN_2_ is shown on the right. Li_2_CN_2_ can be viewed as a hypothetical body-centered (CN_2_)^2−^ lattice whose distorted tetrahedral sites are occupied by Li^+^ [29]. XRD patterns (c) and DRIFT spectra (d) of LiH–C composite samples collected at different reaction temperatures under a N_2_ atmosphere. The XRD and DRIFT spectra of the synthesized Li_2_CN_2_ sample are shown as the reference.

It has been reported that hydrides (H^−^) play a peculiar role in dinitrogen fixation and hydrogenation to ammonia, where the reactive hydridic hydrogen functions as electron and/or hydrogen carriers [26,27]. Our previous investigations show that lithium hydride (LiH) can cleave NN triple bond, forming lithium imide (Li_2_NH). Subsequently, the imide can be hydrogenated to produce NH_3_ [28]. It is interesting to figure out whether the redox chemistry of the Li–N–H system can be extended to N_2_ functionalization beyond ammonia, such as the formation of N–C bonds. As a trial, we initially tested the reaction of Li_2_NH and an easily obtained and straightforward inorganic carbon source, i.e. graphite, at elevated temperatures. It was clearly seen that lithium cyanamide Li_2_CN_2_ composed of [NCN]^2−^ and Li^+^ ions could be obtained (Supplementary Fig. S1), demonstrating the N–C bond formation. This observation inspired us to explore a direct N–C bond formation from N_2_ and graphite mediated by LiH via a one-pot route. Thus, we treated a mixture of LiH and C under a 20-bar N_2_ atmosphere in a homemade reactor at 550ºC for 5 h (Fig. 2b). Only H_2_ was detected as the gaseous product. The solid residue contained Li_2_CN_2_ as the major product, as demonstrated by the X-ray diffraction (XRD) and DRIFT characterizations (red lines of Fig. 2c and d). Bench-scale Li_2_CN_2_ (in grams) was obtained facilely with a yield of >85%. Further amplifying the production by using a larger reactor equipped with an H_2_/N_2_ separation unit would be straightforward. It should be noted that the known methods for Li_2_CN_2_ synthesis typically employ reactive nitrogenous compounds (such as NH_3_, Li_3_N, etc.) and carbon-containing compounds (such as melamine, Li_2_C_2_, CO_2_, etc.) as the N and C sources, and are typically accompanied by the generation of solid byproducts (Fig. 2a) [29–33]. The method developed here has clear advantages in simplicity and atom economy. A step further by feeding the mixture of LiH and C with ^15^N_2_ under 20 bar could also produce ^15^N-labeled Li_2_CN_2_ with high purity (Supplementary Fig. S2), providing a facile and cost-effective new route for ^15^N-labeled C–N-containing raw materials for further organic synthesis.

Albeit that Li_2_CN_2_ formation can be interpreted as an overall reaction of 2LiH + C + N_2_ → Li_2_CN_2_ + H_2_, the course of this gas–solid reaction contains chemistry that is far from well understood. Our temperature-programmed reaction coupled with quasi *in situ* spectroscopy characterization reveals that the H_2_ signal appeared at temperatures of >350ºC showing the occurrence of a redox reaction between H(Li), N_2_ and C (Supplementary Fig. S3). However, only at temperatures of >400ºC could the Li_2_CN_2_ phase and its characteristic C=N vibration (2035 cm^−1^) be observed by XRD and diffuse reflectance infrared Fourier transform spectroscopy (DRIFT) (Fig. 2c and d) [34]. Meanwhile, diffraction peaks at ∼31º and ∼52º, as well as N–H vibration at 3170 cm^−1^ assignable to a kind of solid solution of lithium imide (Li_2_NH) and lithium nitride-hydride (Li_4_NH) (denoted as Li_2+x_NH), appeared [35]. With an increase in temperature, the signals of Li_2+x_NH solid solution fall after rising. We suppose that the hydric H of LiH provides electrons to reduce N_2_ first and converts itself to H_2_, and then the formed solid solution Li_2+x_NH plays a critical role in establishing C–N bonding, i.e. the hydridic H in the solid solution is an electron source for continued N_2_ reduction and perhaps also for the activation of C for subsequent C–N coupling. The involvement of hydride species in carbon reduction has also been identified in previous reports [36].

### Synthesis of carbodiimide using Li_2_CN_2_ as an organic synthon

Lithium cyanamide, Li_2_CN_2_, containing the centrosymmetric [NCN]^2−^ subunits in its crystal structure [29,37], is generally regarded as an inorganic salt and has been used as the source of [NCN]^2−^ subunit to synthesize metal cyanamides in solid-state reactions [38–40]. However, although the [NCN]^2−^ subunit can also be considered to have two cumulated C=N bonds and might be used as an organic building block to synthesize complex nitrogen-containing organic compounds, reports in this area are very rare, probably because Li_2_CN_2_ is commercially unavailable and organic chemists are unfamiliar with this salt.

**Scheme 1.**
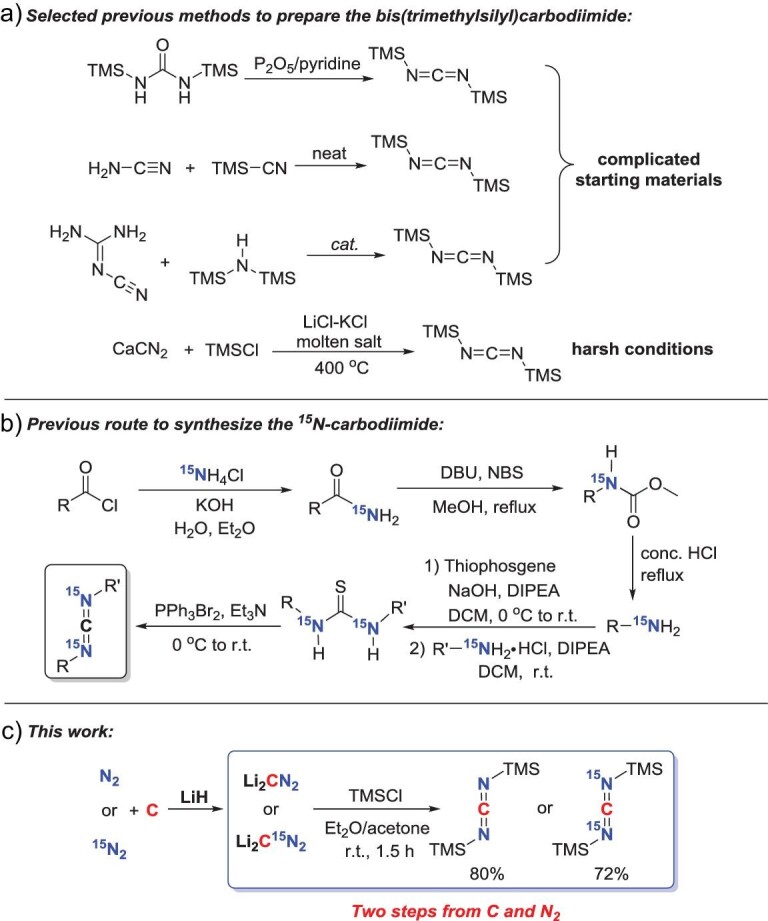
(a) Selected previous methods to prepare the bis(trimethylsilyl)carbodiimide. (b) Previous route to synthesize the ^15^N-carbodiimide. (c) Synthesis of carbodiimide and ^15^N-labeled carbodiimide from N_2_ and carbon (this work). In the meantime, the ^15^N-labeled carbodiimide is a useful NMR probe. For example, it can be used to determine the connectivity and regioregularity of the polycarbodiimides [46]. However, as shown in Scheme 1b, the classic synthetic route of ^15^N-labeled carbodiimide from the commercial ^15^N source ^15^NH_4_Cl is complicated and tedious.

As our above-described process achieved gram-scale (or a much larger amount of) preparation of Li_2_CN_2_ effectively, our strategy to combine the heterogeneous approach with the homogeneous synthetic methodology for N_2_ activation and transformation was halfway successful. With this grand success in hand, we immediately initiated our demonstrative investigation into the synthetic application of Li_2_CN_2_ following the usual homogenous synthetic methodology. Our first attempt was to treat Li_2_CN_2_ simply with Me_3_SiCl (TMSCl), aiming at the synthesis of the corresponding bis(trimethylsilyl)carbodiimide (BTMSC), a useful versatile synthetic intermediate and an important representative carbodiimide derivative. Carbodiimides are a unique class of hetero-cumulene compounds, which are irreplaceable and valuable precursors to synthesize numerous N-heterocycles. Carbodiimide moieties are also present in various drugs and natural products [41]. As illustrated in Scheme 1a, although the BTMSC can be prepared by other methods, such as the dehydration of bis(trimethylsilyl)urea by phosphorus pentoxide [42], the reaction between methylsilyl cyanide and cyanamide [43], or the reaction between dicyandiamide and 1,1,1,3,3,3-hexamethyldisilazane with ammonia sulfate as the catalyst [44], the related reactants in these reactions are complicated. It takes several extra steps to prepare these reagents from more basic raw materials. CaCN_2_ can also react with TMSCl to give the BTMSC, but this reaction needs to be carried out in lithium chloride–potassium chloride molten salt at >400^o^C and the yield is not high due to the large proportion of impurities in the raw material CaCN_2_ [45].

Compared with the reported routes, our two-step synthetic strategy from N_2_ and C to obtain the carbodiimides significantly reduces the energy consumption and the number of steps (Scheme 1c). As shown in Supplementary Table S1, we began our investigation by treating the Li_2_CN_2_ with TMSCl in dry toluene as the solvent under the N_2_ atmosphere at room temperature for 3 h. The corresponding BTMSC was observed in a very low yield (25%, GC yield). Next, the reaction was tested with different solvents such as Et_2_O, THF, acetone, CH_3_CN and hexane at room temperature. Interestingly, the reaction rate was fast when acetone or CH_3_CN was used as the solvent. However, the carbodiimide product will further react with the excessive TMSCl in the system to give a white precipitate, resulting in an overall low yield of BTMSC. Based on these results, we tested the reaction in mixed solvents. When Et_2_O and acetone at 7 : 1 were used as a mixed solvent, the BTMSC could be obtained in high yield (84% GC yield, 80% isolated yield) after 1.5 h at room temperature (Supplementary Table S2). The yield indicated that the reaction could also be used to determine the purity of Li_2_CN_2_.

We next tested the sensitivity of Li_2_CN_2_ to protonic solvents. As shown in Supplementary Table S3, Li_2_CN_2_ was partially hydrolysed when it was soaked in water or alcohol. However, Li_2_CN_2_ was not that sensitive to moisture and air. Li_2_CN_2_ was easy to handle as the raw material and it did not require additional protection during most reactions.

By using ^15^N_2_ gas as the starting material, the corresponding ^15^N-labeled Li_2_CN_2_ can be prepared in good yields. Subsequently, the ^15^N-labeled carbodiimide could be prepared in a similar way and obtained in a high isolated yield (72%). This two-step synthetic method has apparent advantages over traditional ways.

### Synthesis of pyrimidines using Li_2_CN_2_ as an organic synthon

**Scheme 2.**
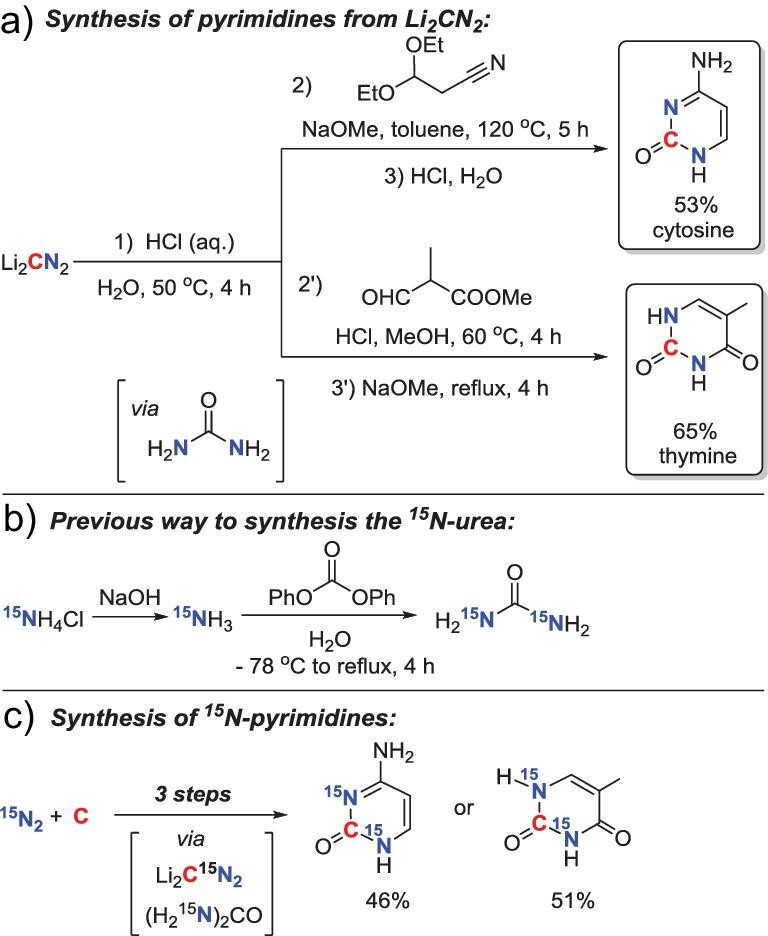
(a) Synthesis of cytosine and thymine from Li_2_CN_2_ via *in situ* generated urea. (b) A previous way to synthesize the ^15^N-urea. (c) Synthesis of ^15^N-labeled pyrimidines via *in situ* generated ^15^N-urea.

With the above successful synthetic application of Li_2_CN_2_, we tried to challenge a more complicated transformation of Li_2_CN_2_ to obtain more value-added nitrogen-containing organic compounds such as pyrimidines, since pyrimidines are not only immediate building blocks for RNA/DNA but also indispensable raw materials in biomedical research and biological drug discovery [23–25]. Cytosine and thymine are two of the pyrimidine bases, which can be prepared based on well-established methods from urea [47–49]. After carefully optimizing the reaction conditions, we found that Li_2_CN_2_ could be hydrolysed into urea *in situ* using HCl aqueous solution within several hours at 50^o^C in high yields (see Supplementary Tables S4 and S5 for more details). It should be noted that urea is unstable to acids and heat. Thus, the heating time and temperature should be controlled for the hydrolysis process. After hydrolysis, water in the reaction bottle was completely evaporated and pyrimidines were then synthesized in the same pot without isolation and purification of urea. As shown in Scheme 2a, following slightly modified known procedures, cytosine was afforded by the condensation of *in situ* generated urea with 3,3-diethoxypropanenitrile and sodium methoxide in toluene, while thymine was prepared by the acid-catalysed condensation of α-formylpropionate with *in situ* generated urea in methanol, followed by an intramolecular cyclization under basic conditions.

The ^15^N-labeled pyrimidines are valuable tools for studying the structural and dynamic features of biomacromolecules. However, the reported ways to synthesize ^15^N-labeled pyrimidines are cumbersome and costly, mainly due to the preparation of ^15^N-urea from the ^15^NH_4_Cl (Scheme 2b) [50]. To prove our strategy, we further expanded the experiments to incorporate ^15^N atoms into the pyrimidines from the ^15^N-labeled Li_2_CN_2_. After hydrolysis of the ^15^N-labeled Li_2_CN_2_ with HCl aqueous solution, the ^15^N-labeled urea could be obtained in a high isolated yield (74%) simply. Without isolation of the ^15^N-labeled urea, the ^15^N-labeled pyrimidines could be obtained successfully following the same procedure as mentioned above (Scheme 2c). These results also confirmed that urea was the intermediate in the reaction and the two N atoms in the pyrimidines were originally from the N_2_ gas.

## CONCLUSIONS

Our homogeneous–heterogeneous synergy strategy for the formation of C–N bonds from N_2_ molecules outlined here differs from those previously described strategies. Typically, forming the C–N bonds from N_2_ requires the reduction of N_2_ to make it nucleophilic enough to react with carbon electrophiles. However, under most conditions, potent reducing agents used in the activation of N_2_ are incompatible with the carbon electrophiles. This incompatibility problem may be solved if the element carbon is used directly as the carbon source instead of using a carbon electrophile. In the first step of our strategy, the key synthetic intermediate Li_2_CN_2_ was obtained with high selectivity by simply reacting carbon, N_2_ gas and LiH. By taking advantage of the heterogeneous approach, the C–N bond was constructed successfully. Whereas Li_2_CN_2_ was primarily reported by inorganic chemists several decades ago, it has been regarded as an inorganic metal salt rather than an organic synthon since then. In the second step of our strategy, we demonstrate that Li_2_CN_2_ can be transformed into complex N-containing organic compounds. The valuable organic precursor bis(trimethylsilyl)carbodiimide was constructed readily from Li_2_CN_2_ in one step. Moreover, cytosine and thymine could also be constructed facilely via Li_2_CN_2_. By using ^15^N_2_ as the feedstock, the corresponding ^15^N-labeled carbodiimide and pyrimidines were also efficiently prepared, which might have a wide range of applications in biochemistry in the future. We believe that our homogeneous–heterogeneous synergy strategy can be used to construct more diversified N-containing organic compounds in the near future.

## MATERIALS AND METHODS

### Preparation of Li_2_CN_2_

The Li_2_CN_2_ sample was prepared by the calcination of ball-milled mixtures of LiH and expanded graphite under 20 bar of N_2_ at 550ºC for 5 h. Feeding the mixture of LiH and expanded graphite with ^15^N_2_ under similar conditions could produce ^15^N-labeled Li_2_CN_2_ with high purity.

### Materials characterization

XRD patterns were recorded on a PANalytical X’pert diffractometer using a homemade sample cell covered with KAPTON film to avoid air contamination. Temperature-programmed reaction experiments were performed in a quartz-lined stainless-steel reactor and the exhaust gases were analysed using an online mass spectrometer (Hiden HPR20). Fourier transform infrared measurements were conducted on a Brucker Tensor II unit in DRIFT mode with a scan resolution of 4 cm^−1^ and an accumulation of 32 scans each time.

### General information for the organic synthesis

Li_2_CN_2_ is stored in a dry and nitrogen-filled glovebox. Tetrahydrofuran (THF) was distilled from sodium-benzophenone in a continuous still under an atmosphere of argon. Toluene, hexane and Et_2_O were purified using an Mbraun SPS-800 solvent purification system. Acetone and acetonitrile were dried over freshly *activated molecular sieves (*4 Å). Other commercially available reagents were used as received without further purification unless otherwise stated. According to the literature procedure, cytosine [47] and thymine [49] were prepared with slight modifications. The synthesis of ^15^N-cytosine and ^15^N-thymine was similar by using Li_2_C^15^N_2_ as the starting material.

## Supplementary Material

nwac168_Supplemental_File

## References

[1] Walter MD . Recent advances in transition metal-catalyzed dinitrogen activation. In: Pedro JP (ed.). Advances in Organometallic Chemistry. New York: Academic Press, 2016, 261–377.

[2] Tanabe Y , Nishibayashi Y. Overviews of the preparation and reactivity of transition metal–dinitrogen complexes. In: Nishibayashi Y (ed.). Transition Metal–dinitrogen Complexes: Preparation and Reactivity. Weinhei: Wiley–VCH, 2019, 1–77.

[3] Rafiqul I , Weber C, Lehmann B et al. Energy efficiency improvements in ammonia production—perspectives and uncertainties. Energy 2005; 30: 2487–504. 10.1016/j.energy.2004.12.004

[4] Chen JG , Crooks RM, Seefeldt LC et al. Beyond fossil fuel-driven nitrogen transformations. Science 2018; 360: eaar6611. 10.1126/science.aar6611 29798857 PMC6088796

[5] Chalkley MJ , Drover MW, Peters JC. Catalytic N_2_-to-NH_3_ (or -N_2_H_4_) conversion by well-defined molecular coordination complexes. Chem Rev 2020; 120: 5582–636. 10.1021/acs.chemrev.9b00638 32352271 PMC7493999

[6] Soloveichik G . Electrochemical synthesis of ammonia as a potential alternative to the Haber–Bosch process. Nat Catal 2019; 2: 377–80. 10.1038/s41929-019-0280-0

[7] Forrest SJ , Schluschaß B, Yuzik-Kilmova EY et al. Nitrogen fixation via splitting into nitrido complexes. Chem Rev 2021; 121: 6522–87. 10.1021/acs.chemrev.0c00958 33973774

[8] Lv Z-J , Wei J, Zhang W-X et al. Direct transformation of dinitrogen: synthesis of N-containing organic compounds via N−C bond formation. Natl Sci Rev 2020; 7: 1564–83. 10.1093/nsr/nwaa142 34691489 PMC8288816

[9] Li J , Yin J, Yu C et al. Direct transformation of N_2_ to N-containing organic compounds. Acta Chim Sinica 2017; 75: 733–43 (in Chinese). 10.6023/A17040170

[10] Kim S , Loose F, Chirik PJ. Beyond ammonia: nitrogen−element bond forming reactions with coordinated dinitrogen. Chem Rev 2020; 120: 5637–81. 10.1021/acs.chemrev.9b00705 32458682

[11] Yang J-H , Peng M, Zhai D-D et al. Fixation of N_2_ into value-added organic chemicals. ACS Catal 2022; 12: 2898–906. 10.1021/acscatal.1c04435

[12] Li D , Zan L, Chen S et al. Direct conversion of N_2_ and O_2_: status, challenge, and perspective. Natl Sci Rev 2022; 9: nwac042.org/10.1093/nsr/nwac042 36726637 PMC9885431

[13] McWilliams SF , Broere DLJ, Halliday CJV et al. Coupling dinitrogen and hydrocarbons through aryl migration. Nature 2020; 584: 221–6. 10.1038/s41586-020-2565-5 32788733 PMC7430000

[14] Zhuo Q , Yang J, Mo Z et al. Dinitrogen cleavage and functionalization with carbon dioxide in a dititanium dihydride framework. J Am Chem Soc 2022; 144: 6972–80. 10.1021/jacs.2c01851 35380823

[15] Xu X , Zhao X, Yang J et al. Direct amination of benzene with molecular nitrogen enabled by plasma-liquid interactions. Angew Chem Int Ed 2022; 61: e202203680. 10.1002/anie.202203680 35332637

[16] Wagner HK , Wadepohl H, Ballmann J. Molybdenum-mediated N_2_-splitting and functionalization in the presence of a coordinated alkyne. Angew Chem Int Ed 2021; 60: 25804–8. 10.1002/anie.202111325 PMC9297880 34618390

[17] Song J , Liao Q, Hong X et al. Conversion of dinitrogen into nitrile: cross-metathesis of N_2_-derived molybdenum nitride with alkynes. Angew Chem Int Ed 2021; 60: 12242–7. 10.1002/anie.202015183 33608987

[18] Wang K , Deng Z-H, Xie S-J et al. Synthesis of arylamines and N-heterocycles by direct catalytic nitrogenation using N_2_. Nat Commun 2021; 12: 248. 10.1038/s41467-020-20270-5 33431885 PMC7801372

[19] Schluschaß B , Borter J-H, Rupp S et al. Cyanate formation via photolytic splitting of dinitrogen. JACS Au 2021; 1: 879–94. 10.1021/jacsau.1c00117 34240082 PMC8243327

[20] Zhong M , Cui X, Wu B et al. Dinitrogen functionalization affording structurally well-defined cobalt diazenido complexes. CCS Chem 2022; 4: 532–9. 10.31635/ccschem.021.202100945

[21] Cui C , Jia Y, Zhang H et al. Plasma-assisted dinitrogen activation via dual platinum cluster catalysis: a strategy for ammonia synthesis under mild conditions. CCS Chem 2022; doi: 10.31635/ccschem.022.202201879 10.31635/ccschem.022.202201879. 10.31635/ccschem.022.202201879

[22] Chen C , Zhu X, Wen X et al. Coupling N_2_ and CO_2_ in H_2_O to synthesize urea under ambient conditions. Nat Chem 2020; 12: 717–24. 10.1038/s41557-020-0481-9 32541948

[23] Pathak T . Azidonucleosides: synthesis, reactions, and biological properties. Chem Rev 2002; 102: 1623–68. 10.1021/cr0104532 11996546

[24] Agrofoglio LA , Gillaizeau I, Saito Y. Palladium-assisted routes to nucleosides. Chem Rev 2003; 103: 1875–916. 10.1021/cr010374q 12744695

[25] Ichikawa E , Kato K. Sugar-modified nucleosides in past 10 years, a review. Curr Med Chem 2001; 8: 385–423. 10.2174/0929867013373471 11172696

[26] Wang Q , Guan Y, Guo J et al. Hydrides mediate nitrogen fixation. Cell Rep Phys Sci 2022; 3: 100779. 10.1016/j.xcrp.2022.100779

[27] Jia H-P , Quadrelli EA. Mechanistic aspects of dinitrogen cleavage and hydrogenation to produce ammonia in catalysis and organometallic chemistry: relevance of metal hydride bonds and dihydrogen. Chem Soc Rev 2014; 43: 547–64. 10.1039/C3CS60206K 24108246

[28] Gao W , Guo J, Wang Q et al. Production of ammonia via a chemical looping process based on metal imides as nitrogen carriers. Nat Energy 2018; 3: 1067–75. 10.1038/s41560-018-0268-z

[29] Down MG , Haley MJ, Hubberstey P et al. Solutions of lithium salts in liquid lithium: preparation and X-ray crystal structure of the dilithium salt of carbodi-imide (cyanamide). J Chem Soc Dalton Trans 1978; 1407–11. 10.1039/dt9780001407

[30] Glaser J , Unverfehrt L, Bettentrup H et al. Crystal structures, phase-transition, and photoluminescence of rare earth carbodiimides. Inorg Chem 2008; 47: 10455–60. 10.1021/ic800985k 18855379

[31] Glaser J , Bettentrup H, Jüstel T et al. Synthesis and properties of tetracyanamidosilicates ARE[Si(CN_2_)_4_]. Inorg Chem 2010; 49: 2954–9. 10.1021/ic902498p 20158217

[32] Hu YH , Huo Y. Fast and exothermic reaction of CO_2_ and Li_3_N into C–N-containing solid materials. J Phys Chem A 2011; 115: 11678–81. 10.1021/jp205499e 21910502

[33] Kimura T , Hotehama C, Sakuda A et al. Mechanochemical synthesis and characterization of amorphous Li_2_CN_2_ as a lithium ion conductor. J Ceram Soc Japan 2019; 127: 518–20. 10.2109/jcersj2.19077

[34] Reckeweg O , Simon A. Azide und cyanamide—ähnlich und doch anders/azides and cyanamides—similar and yet different. Z Naturforsch 2003; 58b: 1097–104. 10.1515/znb-2003-1111

[35] Makepeace JW , Brittain JM, Manghnani AS et al. Compositional flexibility in Li–N–H materials: implications for ammonia catalysis and hydrogen storage. Phys Chem Chem Phys 2021; 23: 15091–100. 10.1039/D1CP02440J 34232235 PMC8294645

[36] Isobe S , Yamada S, Wang Y et al. Microscopic characterization of metal-carbon-hydrogen composites (metal = Li, Mg). J Appl Phys 2013; 114: 093509. 10.1063/1.4820455

[37] Tapia-Ruiz N , Segalés M, Gregory DH. The chemistry of ternary and higher lithium nitrides. Coord Chem Rev 2013; 257: 1978–2014. 10.1016/j.ccr.2012.11.008

[38] Srinivasan R , Ströbele M, Meyer HJ. Chains of [RE_6_] octahedra coupled by (NCN) links in the network structure of RE_2_Cl(CN_2_)N: synthesis and structure of two novel rare earth chloride carbodiimide nitrides with structures related to the RE_2_Cl_3_ type. Inorg Chem 2003; 42: 3406–11. 10.1021/ic020685z 12767174

[39] Kubus M , Glaser J, Kłonkowski A et al. Rare earth carbodiimide silicates: RE_2_(CN_2_)(SiO_4_). Z anorg allg Chem 2010; 636: 991–5. 10.1002/zaac.201000013

[40] Unverfehrt L , Kalmutzki M, Ströbele M et al. Solid state synthesis of homoleptic tetracyanamidoaluminates. Dalton Trans 2011; 40: 9921–4. 10.1039/c1dt10711a 21879080

[41] Wang Y , Zhang W-X, Xi Z. Carbodiimide-based synthesis of N-heterocycles: moving from two classical reactive sites to chemical bond breaking/forming reaction. Chem Soc Rev 2020; 49: 5810–49. 10.1039/C9CS00478E 32658233

[42] Kozyukov VP , Orlov GI, Mironov VF. New methods for the preparation of silylcarbodiimides. Zh Obshch Khim 1981; 51: 245–6.

[43] Mai K , Patil G. An expedient synthesis of bis(trimethylsilyl)carbodiimide. J Org Chem 1987; 52: 275–6. 10.1021/jo00378a020

[44] Mera G , Menapace I, Widgeon S et al. Photoluminescence of as-synthesized and heat-treated phenyl-containing polysilylcarbodiimides: role of crosslinking and free carbon formation in polymer-derived ceramics. Appl Organometal Chem 2013; 27: 630–8. 10.1002/aoc.2993

[45] Stenzel J , Sundermeyer W. Reactions in salt melts. XIV. Preparation of bis-(trimethylsilyl)carbodiimide and bis(trimethylsilyl)acetylene. Chem Ber 1967; 100: 3368–70. 10.1002/cber.19671001027

[46] DeSousa JD , Novak BM. Resolving the regioregularity of poly(N-n-hexyl-N'-phenylcarbodiimide) via nitrogen-15 labeling. ACS Macro Lett 2012; 1: 672–5. 10.1021/mz3001836 35607085

[47] Bendich A , Getler H, Brown GB. A synthesis of isotopic cytosine and a study of its metabolism in the rat. J Biol Chem 1949; 177: 565–70. 10.1016/S0021-9258(18)56999-4 18110434

[48] Sartori G , Maggi R. Acyclic and cyclic ureas. Sci Synth 2005; 18: 665–758.

[49] Guo X , Shen J. An environmentally benign approach to the synthesis of thymine via hydroformylation of methyl acrylate. Monatsh Chem 2014; 145: 657–61. 10.1007/s00706-013-1128-y

[50] Mailyan AK , Chen JL, Li W et al. Short total synthesis of [^15^N_5_]cylindrospermopsins from ^15^NH_4_Cl enables precise quantification of freshwater cyanobacterial contamination. J Am Chem Soc 2018; 140: 6027–32. 10.1021/jacs.8b03071 29672038 PMC6312099

