# Supplementary material for: Synthesis of pyrimidines from dinitrogen and carbon
Source: Natl Sci Rev. 2022 Aug 24;9(12):nwac168. doi: 10.1093/nsr/nwac168 (PMC9905645; doi:10.1093/nsr/nwac168)
Supplement: nwac168_Supplemental_File [file nwac168_supplemental_file.pdf]

Supplementary information

Synthesis of Pyrimidines from Dinitrogen and Carbon

Xianghui Shi[1]†, Qianru Wang[2]†, Chao Qin[2]†, Li-Jun Wu[1], Yuanjin Chen[1], Gao-Xiang Wang[1], Yongli Cai[2,3], Wenbo Gao[2,3], Teng He[2,3], Junnian Wei[1]*, Jianping Guo[2,3]*, Ping Chen[2,3,4]*, Zhenfeng Xi[1]*

[1]Beijing National Laboratory for Molecular Sciences (BNLMS), Key Laboratory of Bioorganic Chemistry and Molecular Engineering of Ministry of Education, College of Chemistry, Peking University, Beijing 100871, China

[2]Dalian Institute of Chemical Physics, Chinese Academy of Sciences, Dalian, 116023, China

[3]University of Chinese Academy of Sciences, Beijing, 100049, China.

[4]State Key Laboratory of Catalysis, Dalian Institute of Chemical Physics, Chinese Academy of Sciences, Dalian, 116023, China

† These authors contributed equally to this work.

*Corresponding author. Email: zfxi@pku.edu.cn, pchen@dicp.ac.cn, jnwei@pku.edu.cn, guojianping@dicp.ac.cn

Table of Contents

Synthesis and characterizations of Li_2CN_2 1

General information for organic synthesis 2

Figure S1 to Figure S21 9

Table S1 to Table S5 30

References 35

Synthesis and characterizations of Li_2CN_2

Li_2CN_2 was prepared via the reaction of LiH and expanded graphite under dinitrogen atmosphere. Typically, LiH (400 mg) and expanded graphite (300 mg) with a LiH to graphite molar ratio of 2:1 were mixed, and ball-milled on a Retsch planetary ball mill (PM 400) at 200 revolutions per minute (r.p.m.) for 10 hours. The black solid residue was heated to 550 °C under a N_2 pressure of 20 bar for 5 hours. The obtained sample was Li_2CN_2. Li_2CN_2 sample was ball-milled in an argon-filled vessel at 200 r.p.m. for 4 hours before being applied for organic synthesis. LiH (99.4% metals basis) was purchased from Alfa Aesar. The expanded graphite was purchased from Aladdin, and treated at 1023 K for 1 minute before use. It should be noted that bench-scale Li_2CN_2 (in grams) could also be obtained facilely with a purity higher than 85%. Further amplifying the production by using an even larger reactor equipped with H_2/N_2 separation unit would be straightforward. By feeding the mixture of LiH and C with $^{15}N_2$ under 20 bar could also produce ^{15}N-labeled Li_2CN_2 with high purity (Fig. S2)

Powder X-ray diffraction patterns were recorded on a PANalytical X'pert diffractometer using a homemade sample cell covered with KAPTON film to avoid air and moisture contamination. Temperature-programmed reaction (TPR) experiments were performed in a quartz-lined stainless steel reactor, and the exhaust gases were analyzed by an online mass spectrometer (Hiden HPR20). Typically, 50 mg sample was heated in a N_2 flow (30 mL min^{-1}) from room temperature to the desired temperature at a ramping rate of 5 K min^{-1}. Fourier transform infrared measurements were conducted on a Brucker Tensor II unit in DRIFT mode with a scan resolution of 4 cm^{-1} and an accumulation of 32 scans each time.

The purity of Li_2CN_2 was not determined by XRD. As XRD technique can only provide the structural information of crystalline phases. Although Li_2CN_2 is the only observed phase, the possibility of some amorphous phases cannot be excluded. The purity of Li_2CN_2 is determined based on the reactions between Li_2CN_2 and HCl aqueous to form urea.

General information for the organic synthesis

Li_2CN_2 is stored in the dry and nitrogen-filled glovebox. Tetrahydrofuran (THF) was distilled from sodium-benzophenone in a continuous still under an atmosphere of argon. Toluene, hexane, and Et_2O were purified using a Mbraun SPS-800 solvent purification system. Acetone and acetonitrile were dried over freshly activated molecular sieves (4 Å). Methyl α-formylpropionate was readily synthesized according to a reported procedure[51]. The procedures of preparing cytosine[47] and thymine[49] were adapted with slight modifications. The yields of cytosine and thymine prepared directly from commercial urea were 72% and 73%, respectively. Other commercially available reagents were used as received without further purification unless otherwise stated.

^1H, ^{13}C, ^{15}N NMR spectra were recorded on a Bruker AVANCE III or a Bruker AVANCE NEO spectrometer. ^1H and ^{13}C NMR spectra were recorded using tetramethylsilane as an internal standard. ^{15}N NMR spectra were referenced externally to $MeNO_2$. Data for NMR were reported as follows: chemical shift (δ, ppm), multiplicities (s = singlet, d = doublet, t = triplet, dd = doublet of doublets, br = broad), coupling constant (J, Hz). IR spectra were recorded with a Bruker Alpha II spectrometer and are reported in wavenumbers (cm^{-1}). High-resolution mass spectra (HRMS) were recorded on a Bruker Solarix XR mass spectrometer using an electrospray ionization (ESI) source. Gas chromatography was carried out with a Shimadzu GC-2014 instrument equipped with a hydrogen flame ionization detector, using N_2 as a carrier gas. The reaction conditions of the synthesis of urea from Li_2CN_2 were determined by reverse-phase HPLC with a Lab Waters Amide column (1.7 μm 2.1*100mm) and a UV detector (195 nm).

Preparation of Bis(trimethylsilyl)carbodiimide

$$Li_2CN_2 + TMSCl \xrightarrow[\text{r. t., 1.5 h}]{Et_2O/\text{Acetone}} TMS{-}N{=}C{=}N{-}TMS$$

TMSCl (402 mg, 3.70 mmol, 2.0 equiv.) was added to a solution of Li_2CN_2 (100 mg, 1.85 mmol, 1.0 equiv.) in a mixed solvent of Et_2O (7.0 mL)/acetone (1.0 mL). The reaction mixture was stirred for 1.5 hours at room temperature. Then the gray turbidity solution was filtered through celite. The filtrate was dried *in vacuo* to afford bis(trimethylsilyl)carbodiimide as a colorless liquid (276 mg, 1.48 mmol, 80 % yield).

^{1}H NMR (600 MHz, C_6D_6): δ 0.10 (br, 18 H, Si*Me*3) ppm. ^{13}C NMR (150 MHz, C_6D_6): δ 0.8 (s, Si*Me*3), 123.4 (s, N*C*N) ppm.

Preparation of ^{15}N-Bis(trimethylsilyl)carbodiimide

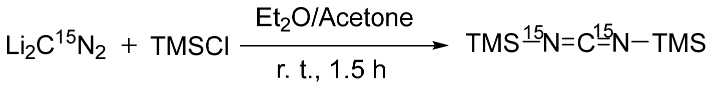

TMSCl (388 mg, 3.57 mmol, 2.0 equiv.) was added to a solution of $Li_2C^{15}N_2$ (100 mg, 1.78 mmol, 1.0 equiv.) in a mixed solvent of Et_2O (7.0 mL)/acetone (1.0 mL). The reaction mixture was stirred for 1.5 hours at room temperature. Then the gray turbidity solution was filtered through celite. The filtrate was dried *in vacuo* to afford ^{15}N-bis(trimethylsilyl)carbodiimide as a colorless liquid (242 mg, 1.28 mmol, 72 % yield).

^{1}H NMR (600 MHz, C_6D_6): δ 0.15 (br, 18 H, Si*Me*3) ppm. ^{13}C NMR (150 MHz, C_6D_6): δ 0.8 (s, Si*Me*3), 123.4 (t, ^{15}N*C*^{15}N) ppm. ^{15}N NMR (61 MHz, C_6D_6): δ -331.0 (s) ppm. HRMS: *m/z*: calcd for $C_7H_{18}{}^{15}N_2Si_2$ $[M+H]^+$: 189.10220, found: 189.10209. IR: 2960 (m), 2201 (s), 1252 (m), 838 (s), 734 (m) cm^{-1}.

Preparation of Urea from Li_2CN_2

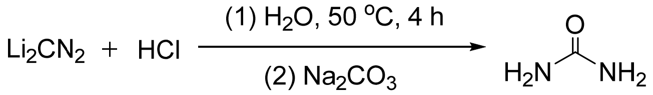

Li_2CN_2 (108 mg, 2.00 mmol, 1.0 equiv.) was added to a 10 mL vial that contains a magnetic stirring bar, then 6.7 mL HCl solution (1.5 mol/L in water, 10 mmol, 5.0 equiv.) was added. The reaction vial was sealed with a cap, heated to 50 °C, and stirred for 4 hours. After cooling to room temperature, the mixture was neutralized with a solution of Na_2CO_3 (1.5 mol/L in water). The black residue was removed, and the filtrate was dried *in vacuo* to yield urea as a white solid. Because both salt and urea are soluble in water, there is no way to remove all the salt in this step. The yield of urea (1.74 mmol, 87 % yield) was calculated by ^{1}H and quantitative ^{13}C NMR with the benzophenone used as the internal standard.

^{1}H NMR (600 MHz, DMSO-d_6): δ 5.43 (br, 4 H, N*H*2) ppm. ^{13}C NMR (150 MHz, DMSO-d_6): δ 160.1 (s, *C*=O) ppm.

Preparation of ^{15}N-Urea

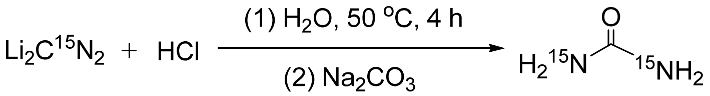

$Li_2C^{15}N_2$ (86 mg, 1.53 mmol, 1.0 equiv.) was added to a 10 mL vial that contains a magnetic stirring bar, then 5.1 mL HCl solution (1.5 mol/L in water, 7.6 mmol, 5.0 equiv.) was added. The reaction vial was sealed with a cap, heated to 50 °C, and stirred for 4 hours. After cooling to room temperature, the mixture was neutralized with a solution of Na_2CO_3 (1.5 mol/L in water). The black residue was removed, and the filtrate was dried *in vacuo* to yield ^{15}N-urea as a white solid. Because both salt and urea are soluble in water, there is no way to remove all the salt in this step. The yield of urea (1.13 mmol, 74 % yield) was calculated by ^{1}H and quantitative ^{13}C NMR with the benzophenone used as the internal standard.

^{1}H NMR (600 MHz, DMSO-d$_6$): 5.71, 5.86 (d, J = 87 Hz, 4 H, $^{15}NH_2$) ppm. ^{13}C NMR (150 MHz, DMSO-d$_6$): 161.0 (t, C^{15}N) ppm. ^{15}N NMR (61 MHz, DMSO-d$_6$): δ - 303.2 (t, J = 89 Hz) ppm. IR: 3455 (s), 1647 (s), 1478 (s), 1149 (m), 768 (m), 603 (s) cm^{-1}.

Preparation of Cytosine

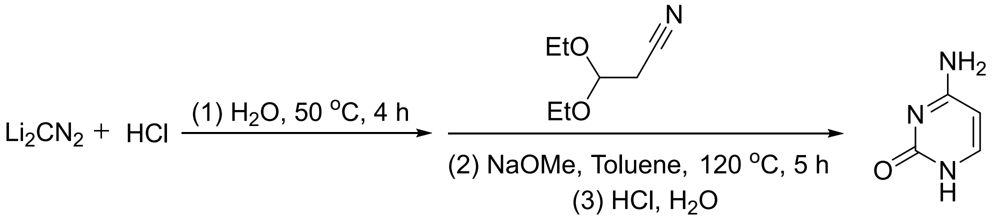

According to the literature procedure[47], cytosine was prepared with slight modifications. Li_2CN_2 (162 mg, 3.01 mmol, 1.0 equiv.) was added in a 50 mL three-necked flask with a magnetic stirring bar, then 10 mL diluted HCl solution (1.5 mol/L in water, 15 mmol, 5.0 equiv.) was added and the mixture was stirred for 4 hours at 50 °C. Then the reaction mixture was carefully concentrated *in vacuo* to remove all the remaining water. A solution of NaOMe (486 mg, 9.00 mmol, 3.0 equiv.) in 15 mL toluene was added under the N_2 atmosphere, and the flask was equipped with a condenser. The mixture was stirred for 0.5 hour at 75 °C to allow the reaction component to dissolve. Then the 3,3-diethoxypropanenitrile (859 mg, 6.00 mmol, 2.0 equiv.) was added. After that, the resulting mixture was heated to 120 °C and refluxed for 5 hours. Vigorously

stirring was necessary during this reaction. Upon completion of the reaction, the solution was concentrated to dryness *in vacuo*, and the residual was dissolved with 3 mL 10 % HCl solution. After stirring for 2 hours at 50 °C, the acid solution was neutralized with ammonium hydroxide. At the end of the reaction, the mixture was filtered through celite, and the filtrate was dried *in vacuo*. The cytosine was purified through a silica gel column with DCM/MeOH (v/v = 3/1) and obtained as a white solid (177 mg, 1.59 mmol, 53 % yield). It should be noted that cytosine did not dissolve well in organic solvents, so a portion was lost during the column purification.

^{1}H NMR (600 MHz, DMSO-d$_6$): δ 5.59 (d, J = 7 Hz, 1 H, NCHC*H*), 7.08 (br, 2 H, N*H*$_2$), 7.34 (d, J = 7 Hz, 1 H, NC*H*), 10.55 (br, 1 H, N*H*) ppm. ^{13}C NMR (150 MHz, DMSO-d$_6$): δ 93.0 (s, NCH*C*H), 143.1 (s, NH$_2$), 157.4 (s, *C*O), 167.2 (s, N*C*H) ppm.

Preparation of ^{15}N-Cytosine

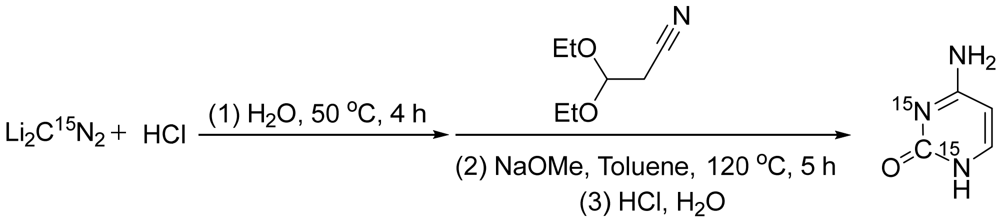

The synthesis of ^{15}N-cytosine was similar to the procedure mentioned above. The $Li_2C^{15}N_2$ (112 mg, 2.00 mmol, 1.0 equiv.) was used as the starting material. The ^{15}N-cytosine was obtained as a white solid (104 mg, 0.92 mmol, 46 % yield) after the column purification.

^{1}H NMR (600 MHz, DMSO-d$_6$): δ 5.58, 5.60 (dd, J = 3.36, 3.36 Hz, 1 H, NCHC*H*), 7.32, 7.33 (dd, J = 2.76, 2.88 Hz, 1 H, NC*H*) ppm. ^{13}C NMR (150 MHz, DMSO-d$_6$): δ 93.0 (s, NCH*C*H), 143.1 (d, *C*NH$_2$), 157.3, 157.4 (dd, *C*O), 167.0 (s, N*C*H) ppm. ^{15}N NMR (61 MHz, DMSO-d$_6$): δ - 240.8 (s), -168.6 (s) ppm. HRMS: *m/z*: calcd for $C_4H_5{}^{15}N_2N_1O_1$ [M+H]$^+$: 114.04461, found: 114.04448. IR: 3330 (s), 3090 (s), 2805 (s), 1663 (m), 1447 (m), 1387 (m), 1223 (s), 783 (m), 578 (m) cm^{-1}.

Preparation of Thymine

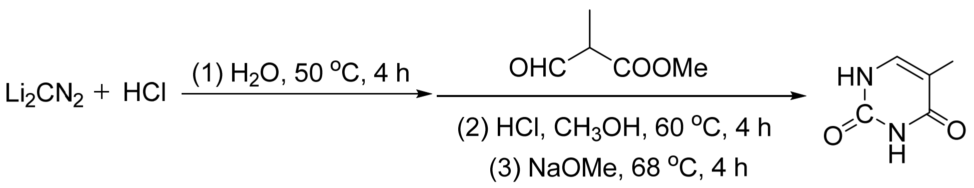

According to the literature procedure[49], thymine was prepared with slight modifications. Li_2CN_2 (180 mg, 3.33 mmol, 1.0 equiv.) was added in a 50 mL three-necked flask with a magnetic stirring bar, then 11 mL diluted HCl solution (1.5 mol/L in water, 16.5 mmol, 5.0 equiv.) was added, and the mixture was stirred for 4 hours at 50 °C. Then the reaction mixture was carefully concentrated *in vacuo* to remove all the remaining water. A solution of the methyl α-formylpropionate (773 mg, 6.66 mmol, 2.0 equiv.) and concentrated HCl (0.2 mL) in 20 mL methanol was added under the N_2 atmosphere. The flask was equipped with a condenser. The reaction mixture was heated to 60 °C and stirred for 4 hours. After that, NaOMe (489 mg, 9.06 mmol, 2.7 equiv.) was added to the mixture. The resulting solution was heated to 68 °C for another 4 hours. After cooling to room temperature, the mixture was acidified with 3 mL 10% HCl solution, then dried *in vacuo*. The thymine was separated through the silica gel column with PE/MeOH (v/v = 5/1) and obtained as a white solid (273 mg, 2.16 mmol, 65 % yield). It should be noted that thymine did not dissolve well in organic solvents, so a portion was lost during the column purification.

^{1}H NMR (600 MHz, DMSO-d_6): δ 1.73 (s, 3 H, CH_3), 7.25 (d, J = 4 Hz, 1 H, NHCH), 10.57 (s, 1 H, NHCH), 10.99 (br, 1 H, CONHCO) ppm. ^{13}C NMR (150 MHz, DMSO-d_6): δ 12.3 (s, CH$_3$), 108.1 (s, CCH$_3$), 138.2 (s, CHNH), 152.0 (s, NHCONH), 165.4 (s, COCCH$_3$) ppm.

Preparation of ^{15}N-Thymine

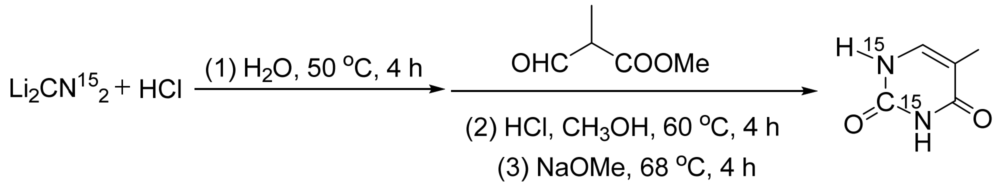

The synthesis of complex ^{15}N-cytosine was similar to the procedure mentioned above. The $Li_2C^{15}N_2$ (83 mg, 1.48 mmol, 1.0 equiv.) was used as the starting material. The ^{15}N-thymine was obtained as a white solid (97 mg, 0.76 mmol, 51 % yield).

^{1}H NMR (600 MHz, DMSO-d_6): δ 1.73 (s, 3 H, CH_3), 7.25 (d, J = 2.4 Hz, 1 H, ^{15}NHCH), 10.58 (br, 1 H, ^{15}NHCH), 10.92, 11.07 (d, J = 90 Hz, 1 H, OC^{15}NHCO) ppm. ^{13}C NMR (150 MHz, DMSO-d_6): δ 12.3 (s, CH$_3$), 108.1 (d, CCH$_3$), 138.2 (d, ^{15}NHCH), 151.9, 152.0 (dd, ^{15}NHCO^{15}NH), 165.4 (d, COCCH$_3$) ppm. ^{15}N NMR (61 MHz, DMSO-d_6): δ - 252.5 (s), - 224.2 (s) ppm. HRMS: *m/z*: calcd for $C_5H_6{}^{15}N_2O_2$ [M+H]$^+$: 129.04427, found: 129.04426. IR: 3202 (s), 3063 (s), 2929 (s), 1732 (s), 1676 (s), 1447 (m), 1210 (s), 849 (s), 813 (m), 763 (m) cm^{-1}.

The reaction between Li_2CN_2 and TMSCl detected by GC

GC running was performed using a Shimadzu GC-2014 instrument, fitted with a TC-FFAP capillary column (30 m×0.25 mm I.D., 0.25 μm film thickness). GC conditions were as follows: injector and detector at 240 ℃; carrier gas, nitrogen (N_2); flame ionization detection (FID); precolumn pressure of 95 kPa. The column temperatures were programmed as follows: 40 ℃ (hold for 2 min), then heated to 250 ℃ at a constant rate of 20 ℃ min^{-1}.

The standard curve was established by using 10.0-100.0 mg pure (99 %) bis(trimethylsilyl)carbodiimide (BTMSC) and 40 μL dodecane as an internal standard. Plots of peak area ratio (response) against newly-formed BTMSC from the reaction of Li_2CN_2 (20 mg, 0.37 mmol, 1.0 equiv.) with TMSCl (81 mg, 0.74 mmol, 2.0 equiv.) were used. The effects of different solvents for BTMSC conversion at room temperature are shown in Table S1.

Stability of Li_2CN_2 to moisture and protonic solvents

The sensitivity of Li_2CN_2 was measured by determining the yield of BTMSC from Li_2CN_2 after being treated with protonic solvents (H_2O, MeOH, and EtOH) or exposed to air (Table S3) in the optimized procedure.

Synthesis of urea from Li_2CN_2

The synthesis of urea from Li_2CN_2 was recorded with the reverse-phase HPLC equipped with a Flaxar DAD diode array detector (column: Waters Amide 1.7 μm column, 2.1 mm × 100 mm; gradient eluent used for separation (A) acetonitrile and (B) water: 0-2 min, 95%; 2-4 min, 95%-60%; 4-6 min, 60%; 6-10 min, 95%; flow rate: 0.4 mL min^{-1}; column oven temperature: 30 ℃; injection volume: 5 μL; diode array detector: 195 nm). The standard curve was established by using 20, 50, 100, 200, 400, 600, 800 μg mL^{-1} pure urea (acetonitrile solution) as the standard.

Sample preparation: Li_2CN_2 (32.4 mg, 0.60 mmol, 1.0 equiv.) reacted with different equivalents of diluted HCl solution (3.0 equiv., 5.0 equiv. and 7.0 equiv.) in a 10 mL sealed vial with a magnetic stirring bar, and the final total volume was 2.0 mL. After stirring for a certain time at different temperatures, 100 μL of the solution was taken out precisely and neutralized to pH = 7 with Na_2CO_3 solution (0.75 mol/L in water), and then diluted to 1.0 mL with the mobile phase solution. Subsequently, 200 μL solution was taken out precisely and diluted into 800 μL with the

mobile phase solution. Before the sample was injected into the HPLC column, it was filtered through a 0.22 μm porosity filter. It should be noted that the yields shown in Table S4 and Table S5 were only used to determine the hydrolysis conditions. The numbers in Table S4 and S5 were not accurate yields, because the impurities in Li_2CN_2 could affect the UV absorption and make the values over 100% in some cases.

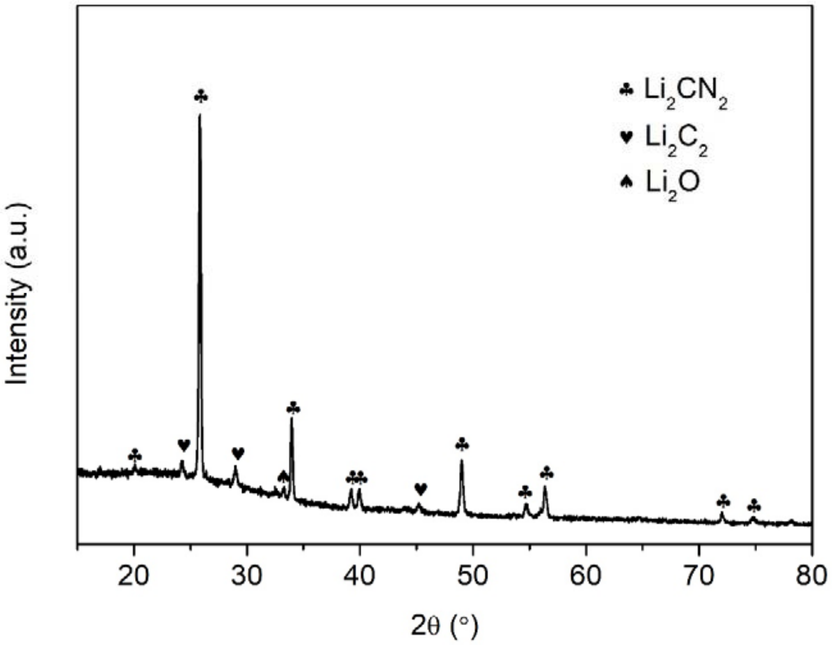

Figure S1 XRD pattern of the Li_2NH-C composite sample collected at 550 °C under argon atmosphere.

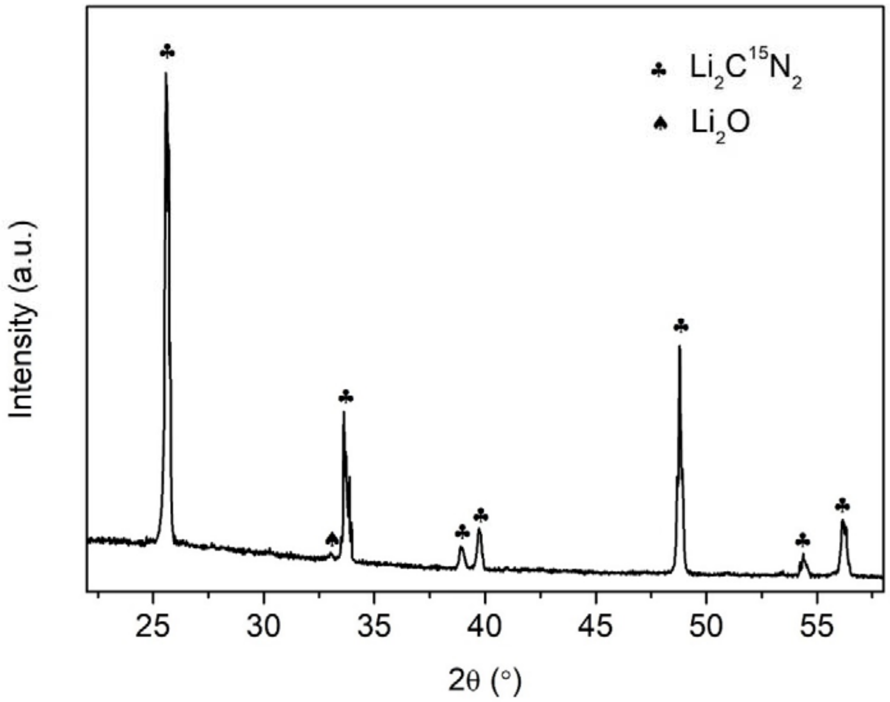

Figure S2 XRD pattern of LiH-C composite sample collected at 550 °C under 20 bar $^{15}N_2$ atmosphere.

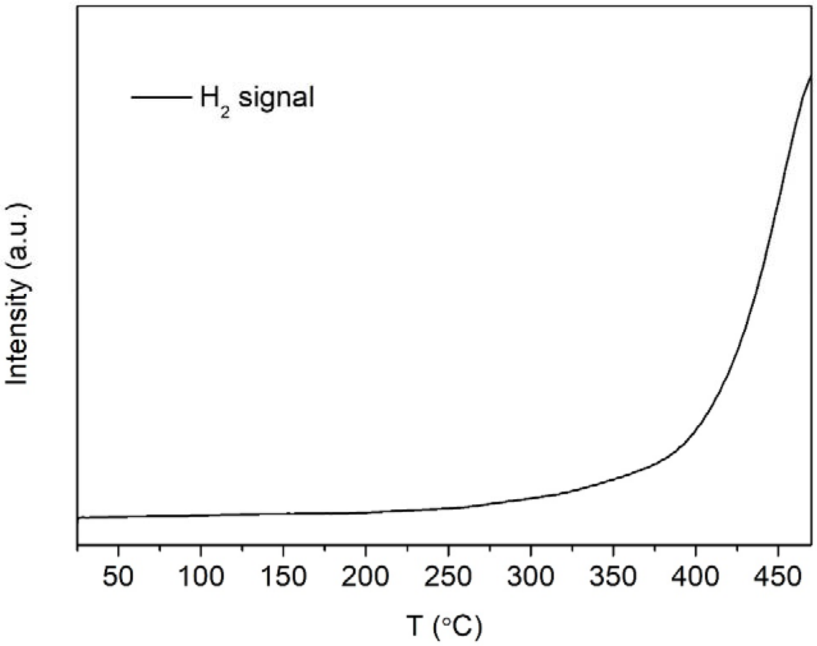

Figure S3 N_2-TPR-MS profile of LiH-C composite sample.

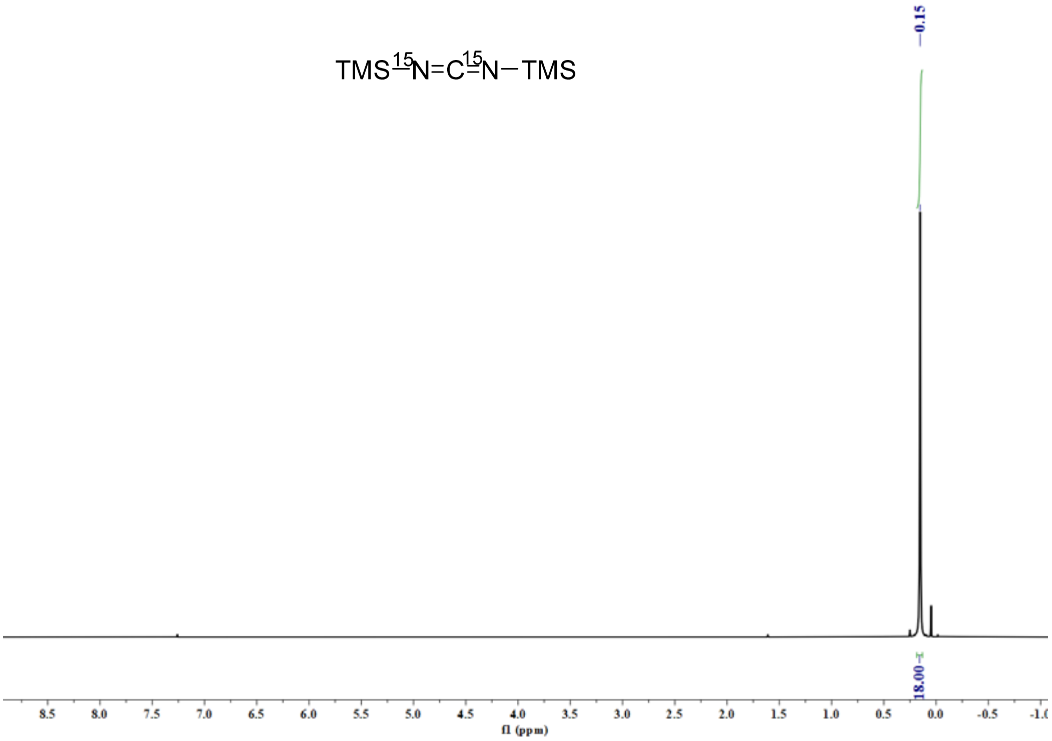

Figure S4 ^{1}H NMR spectrum (600 MHz) of ^{15}N-Bis(trimethylsilyl)carbodiimide in C_6D_6.

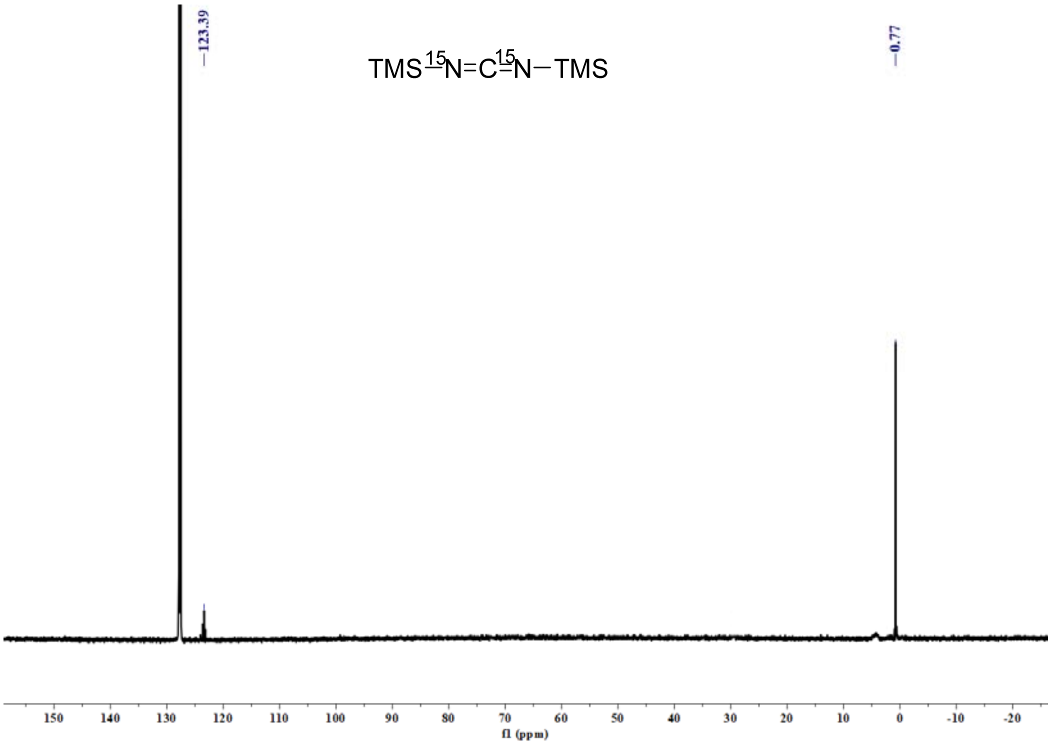

Figure S5 ^{13}C NMR spectrum (150 MHz) of ^{15}N-Bis(trimethylsilyl)carbodiimide in C_6D_6.

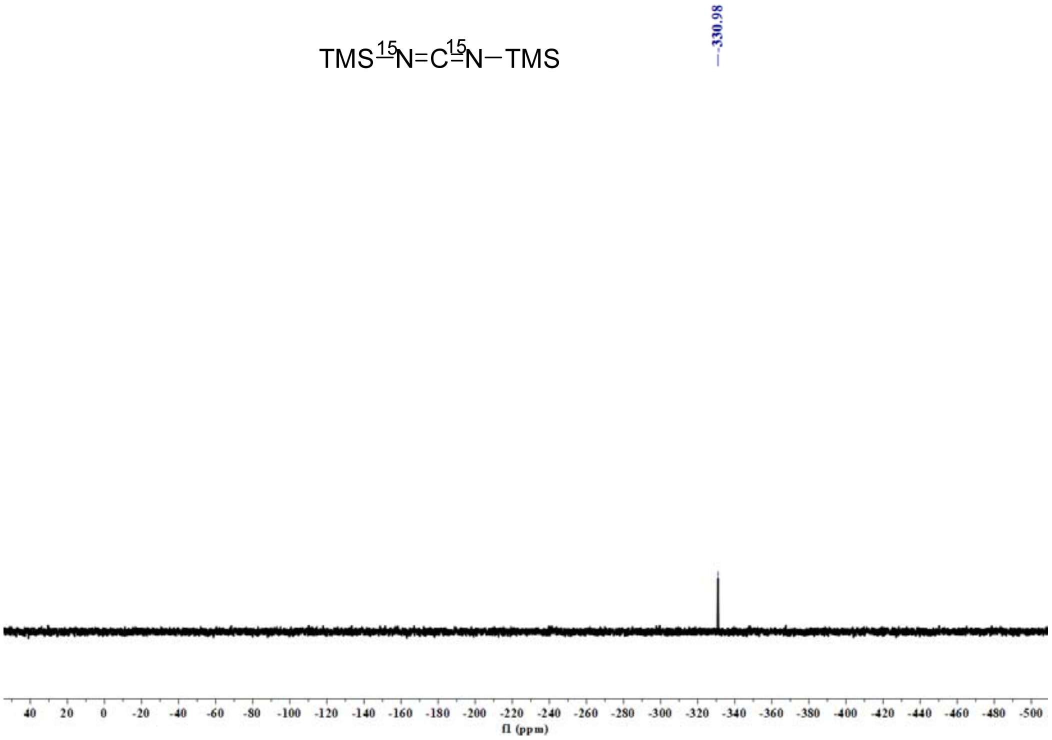

Figure S6 ^{15}N NMR spectrum (61 MHz) of ^{15}N-Bis(trimethylsilyl)carbodiimide in C_6D_6.

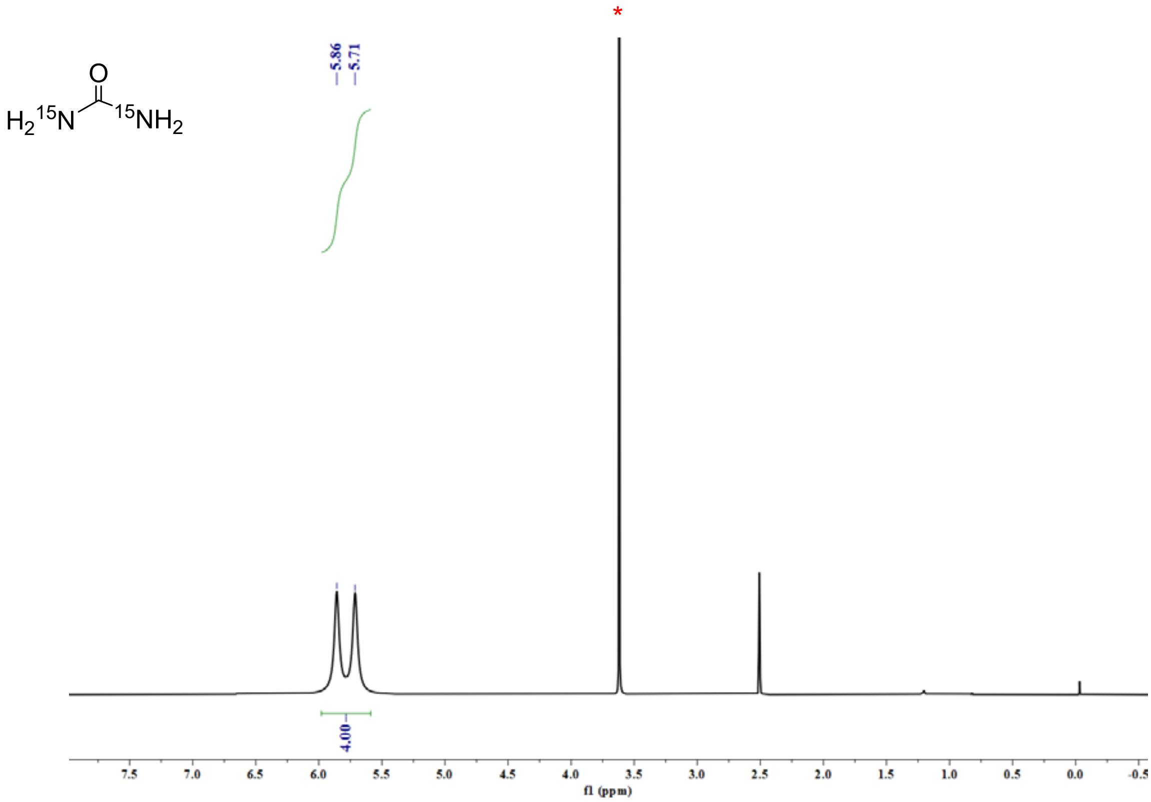

Figure S7 ^{1}H NMR spectrum (600 MHz) of ^{15}N-Urea in DMSO-d_6. (* = H_2O)

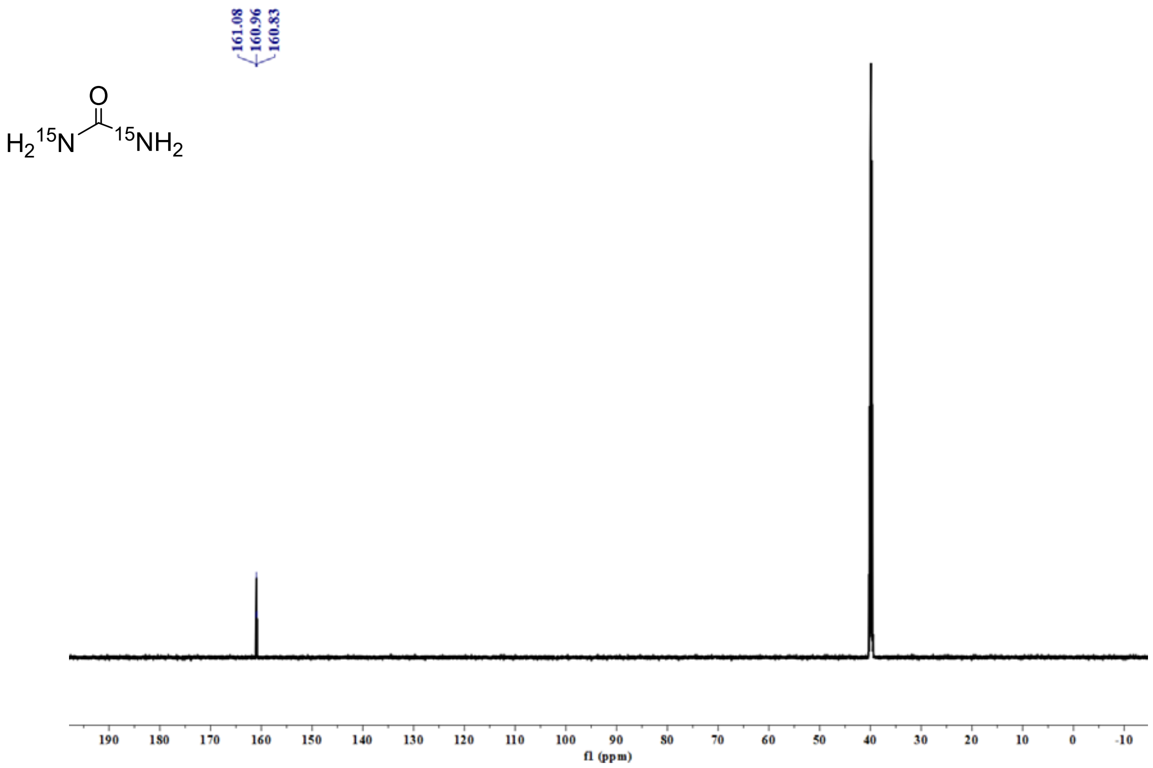

Figure S8 ^{13}C NMR spectrum (150 MHz) of ^{15}N-Urea in DMSO-d_6.

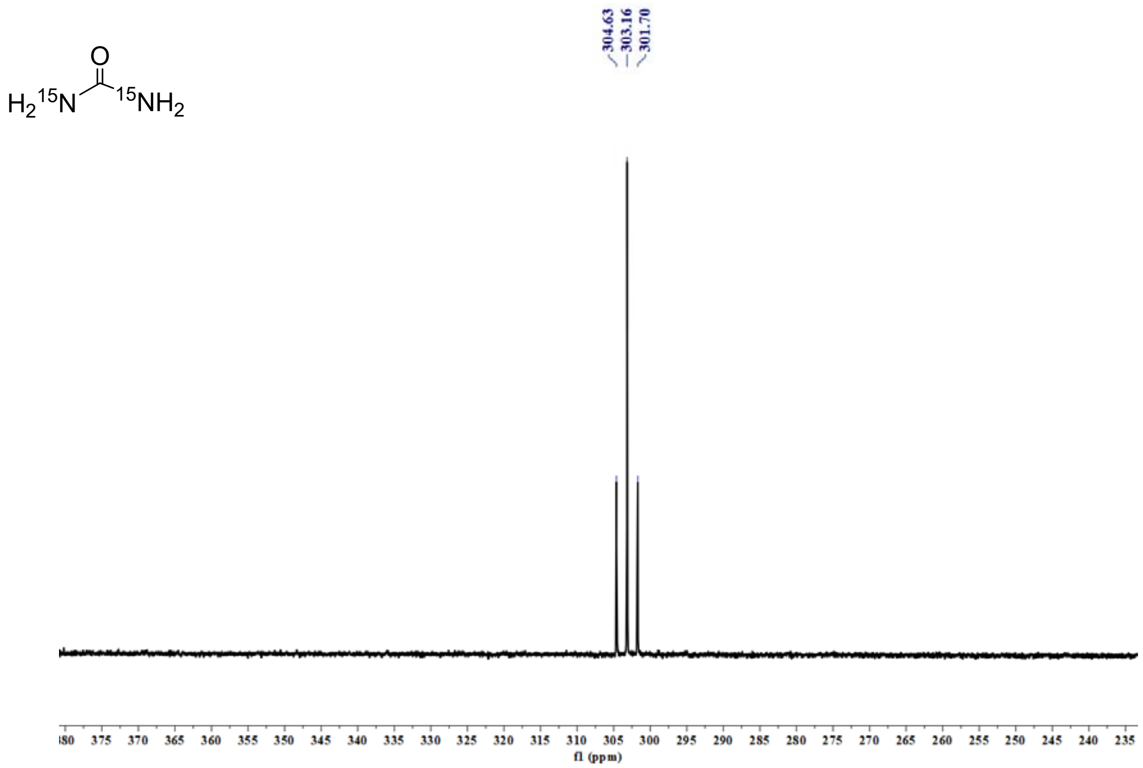

Figure S9 ^{15}N NMR spectrum (61 MHz) of ^{15}N-Urea in DMSO-d_6.

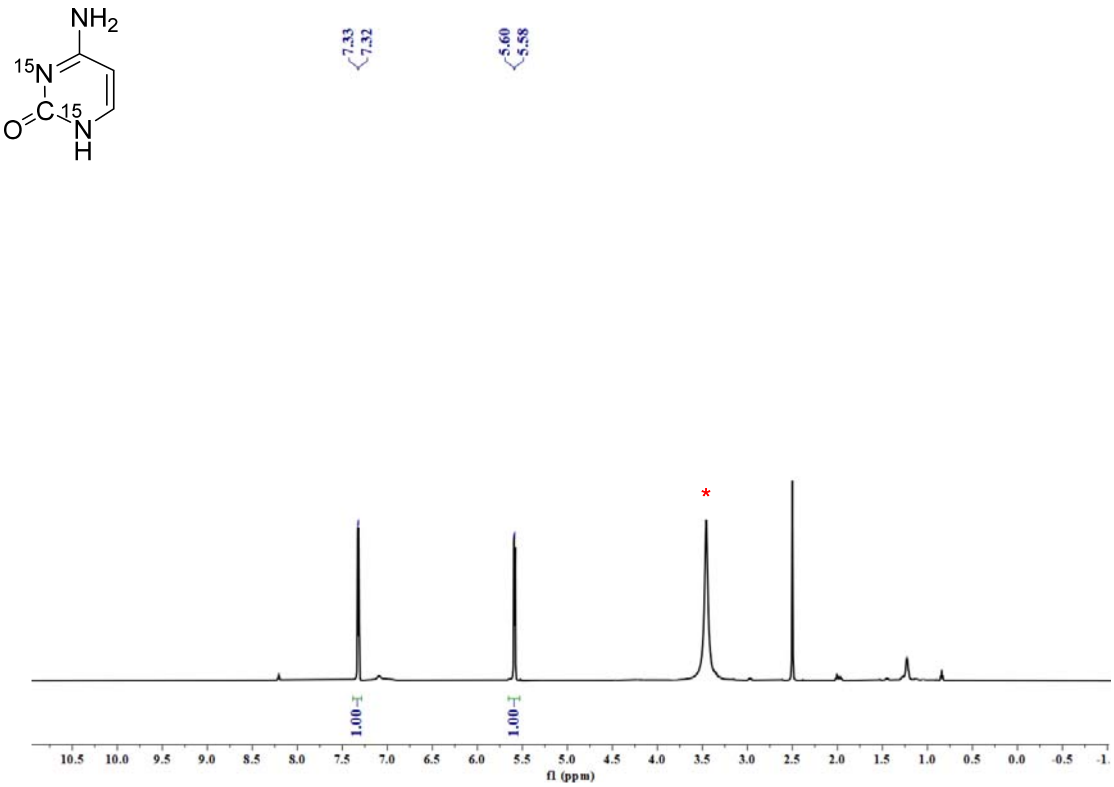

Figure S10 ^{1}H NMR spectrum (600 MHz) of ^{15}N-Cytosine in DMSO-d_6. (* = H_2O)

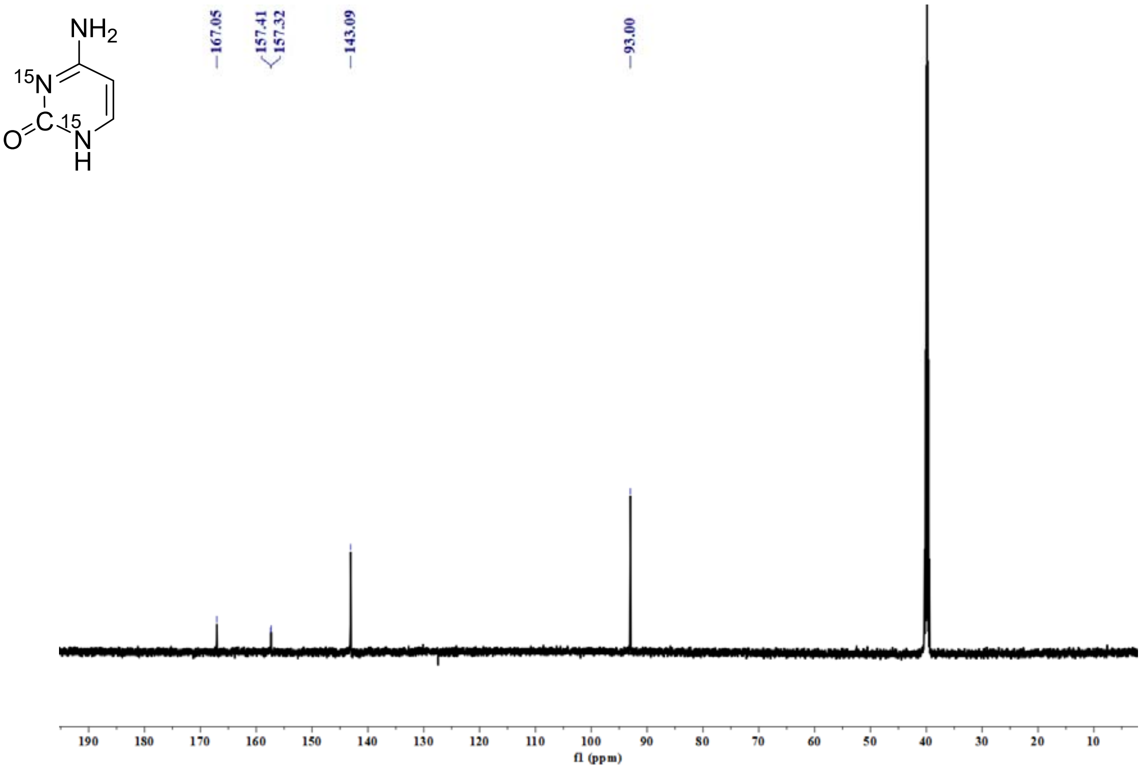

Figure S11 ^{13}C NMR spectrum (150 MHz) of ^{15}N-Cytosine in DMSO-d_6.

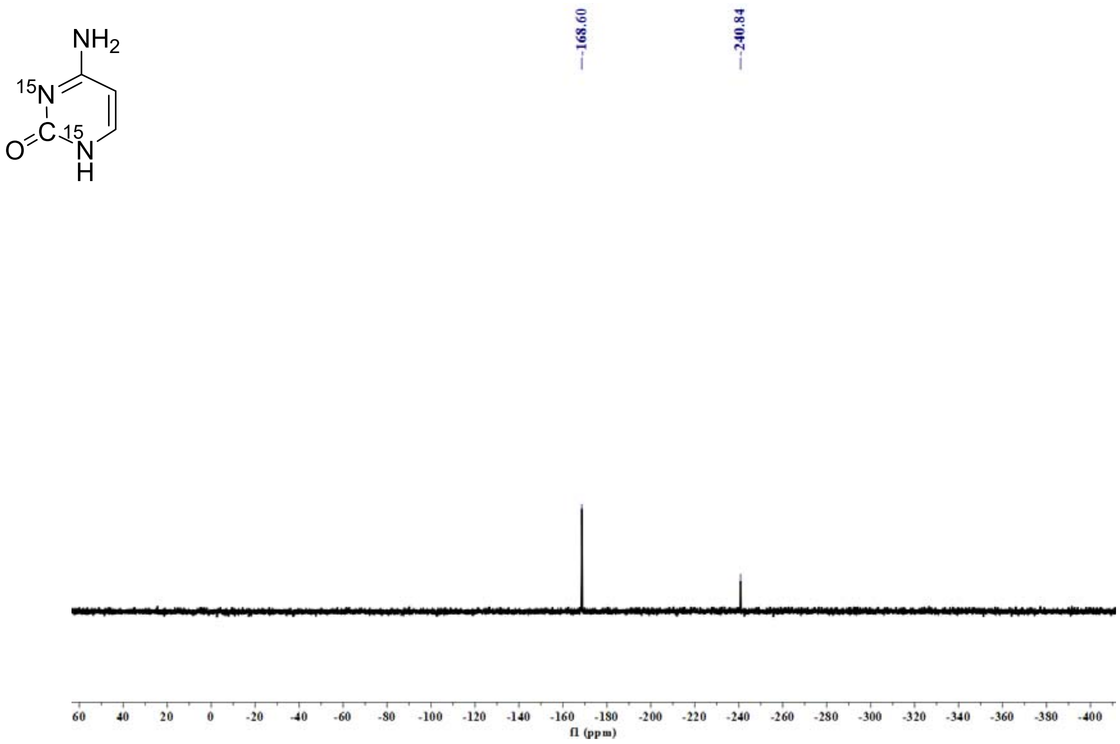

Figure S12 ^{15}N NMR spectrum (61 MHz) of ^{15}N-Cytosine in DMSO-d_6.

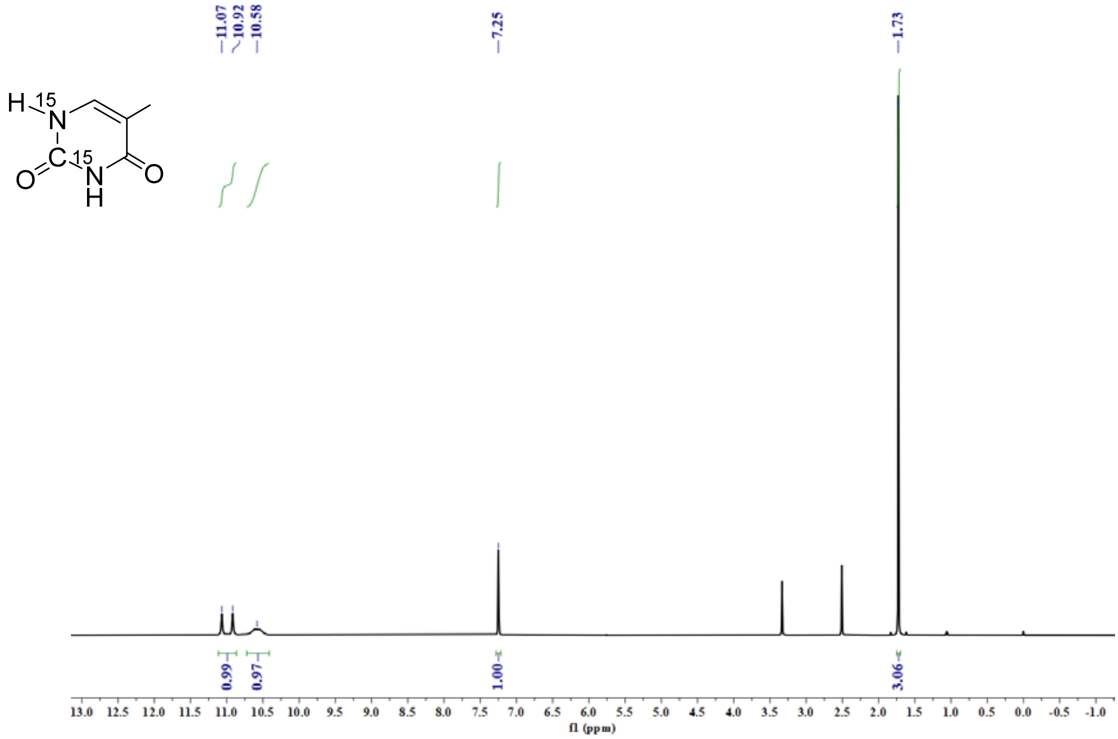

Figure S13 ^{1}H NMR spectrum (600 MHz) of ^{15}N-Thymine in DMSO-d_6.

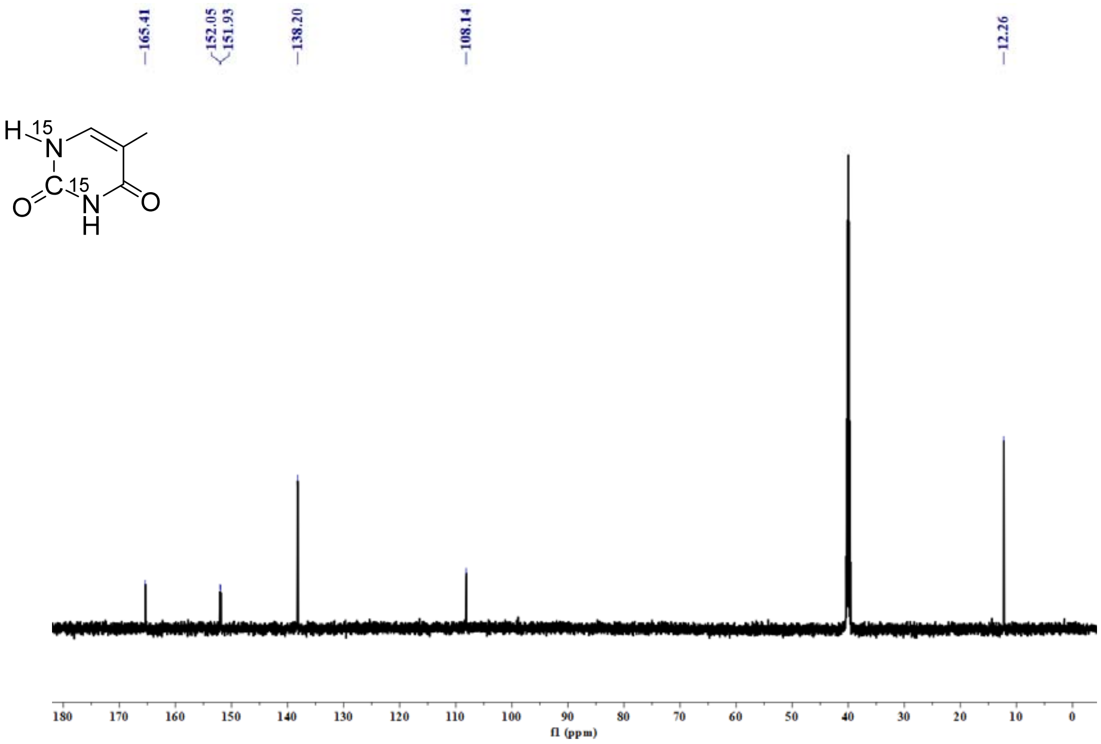

Figure S14 ^{13}C NMR spectrum (150 MHz) of ^{15}N-Thymine in DMSO-d_6.

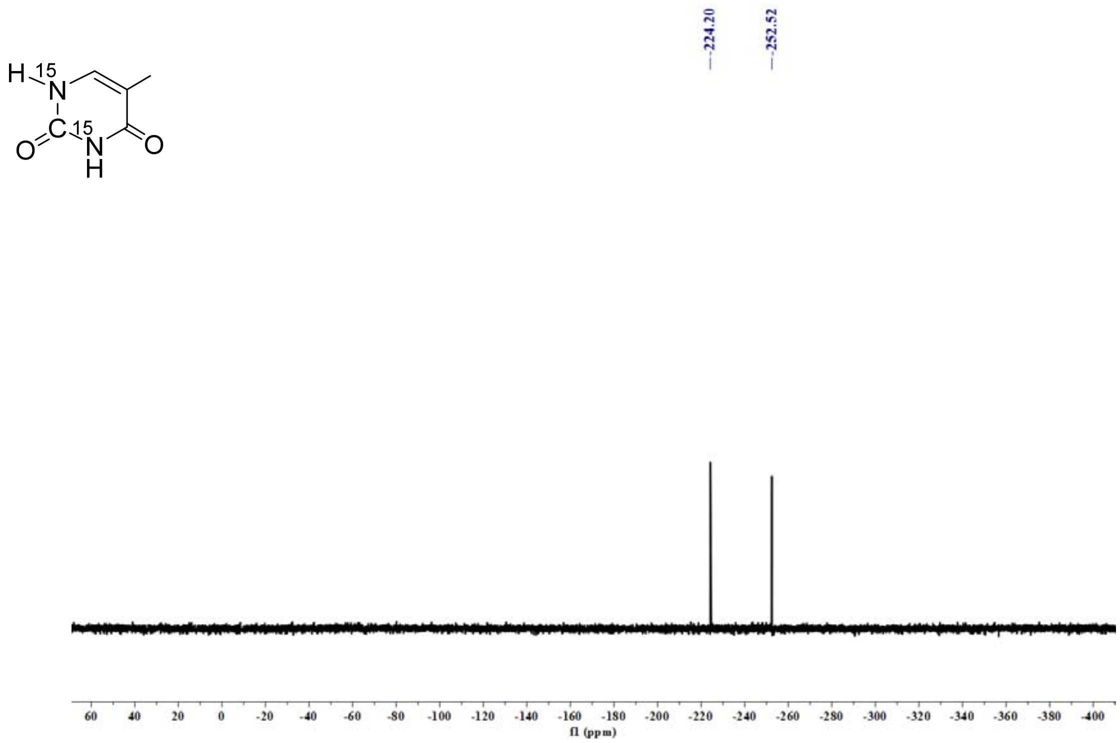

Figure S15 ^{15}N NMR spectrum (61 MHz) of ^{15}N-Thymine in DMSO-d_6.

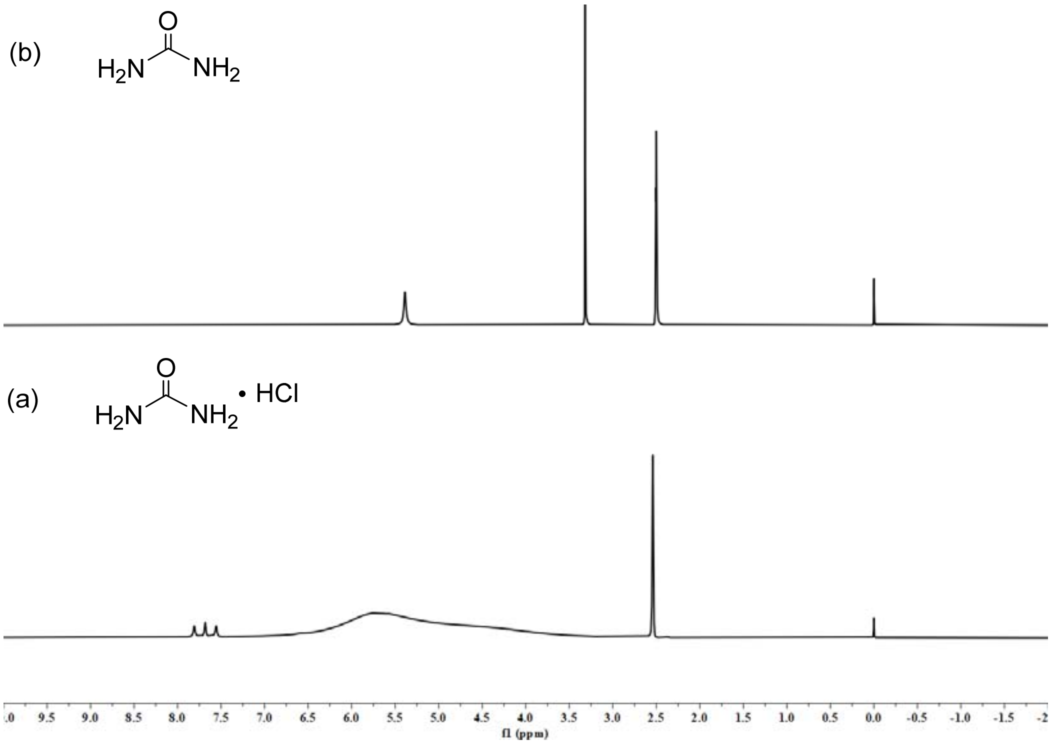

Figure S16 Stacked ^{1}H NMR spectrum (400 MHz) of (a) *in situ* reaction between Li_2CN_2 and HCl aqueous to form urea hydrochloride (broad resonance is assigned to proton of HCl and disappears after neutralization) and (b) neutralization reaction with Na_2CO_3 to yield urea in DMSO-d_6.

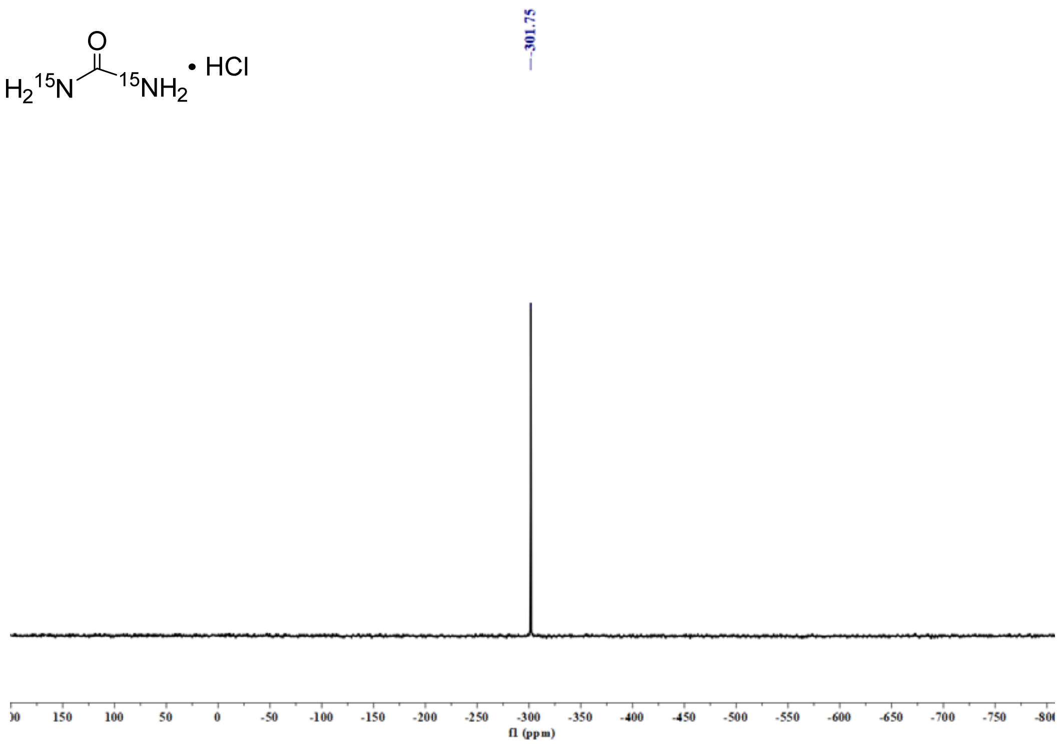

Figure S17 ^{15}N NMR spectrum (61 MHz) of ^{15}N- urea hydrochloride obtained from *in situ* reaction between $Li_2C^{15}N_2$ and HCl in H_2O.

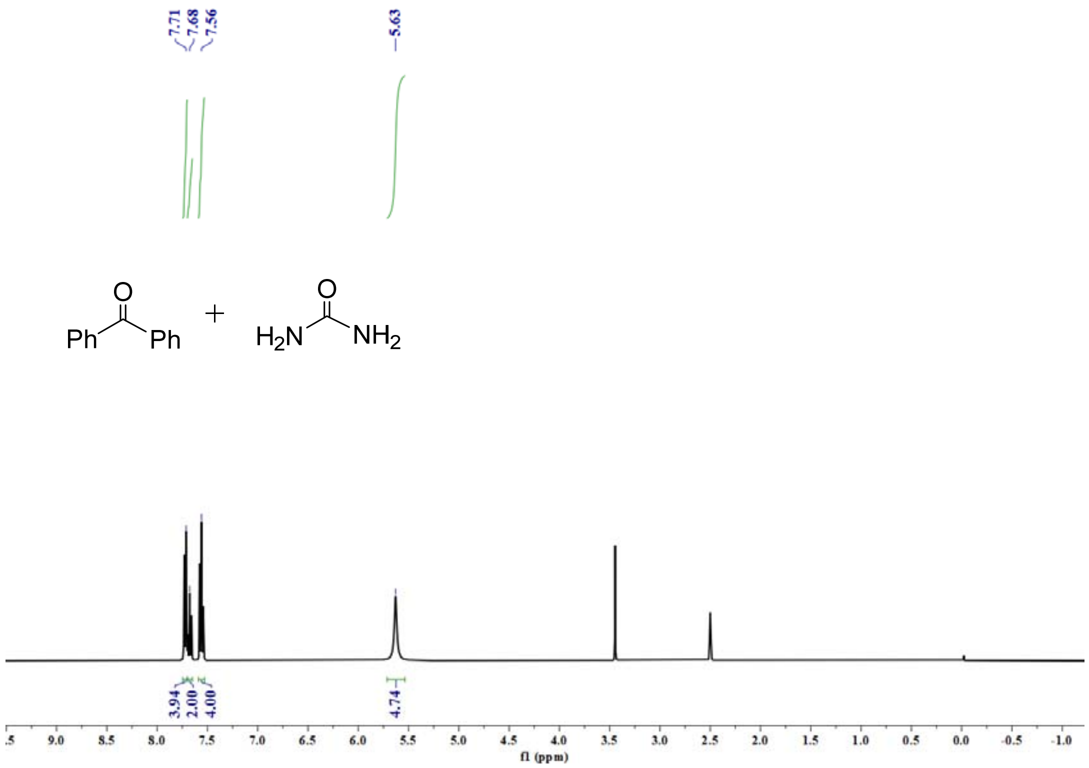

Figure S18 ^{1}H NMR spectrum (600 MHz) of urea (0.071 mmol) and an internal standard of benzophenone (0.052 mmol) in DMSO-d_6 for the determination of the yield of urea.

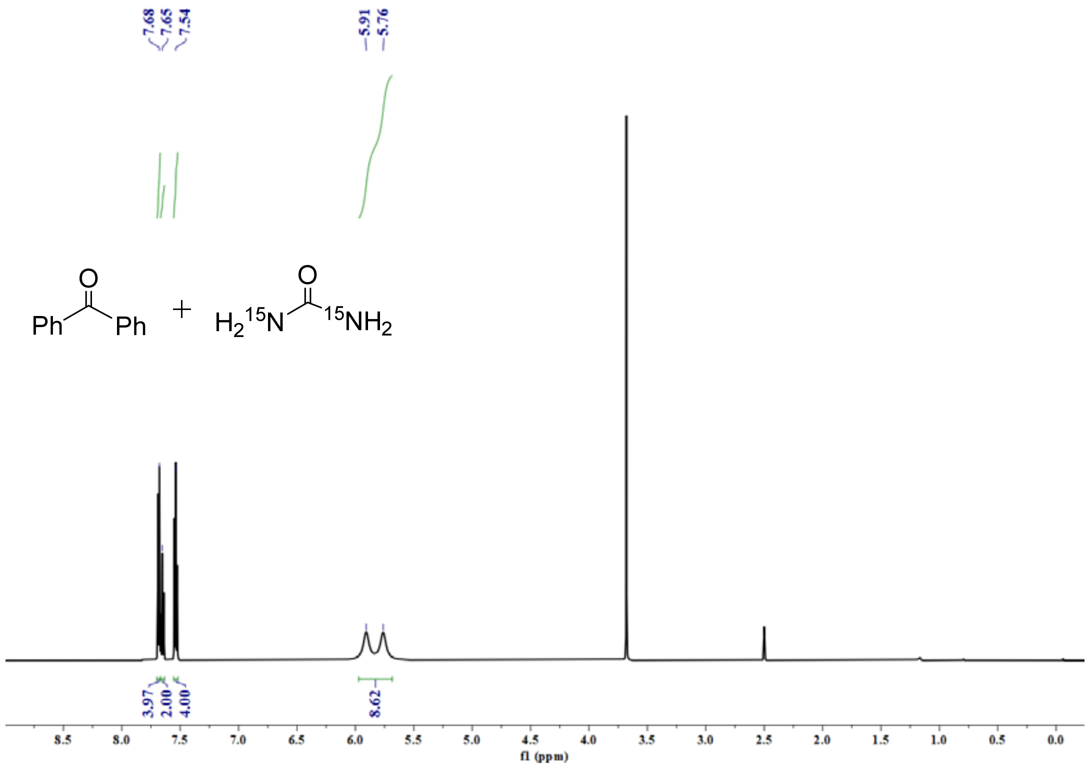

Figure S19 ^1H NMR spectrum (600 MHz) of ^{15}N-urea (0.082 mmol) and an internal standard of benzophenone (0.028 mmol) in DMSO-d_6 for the determination of the yield of urea.

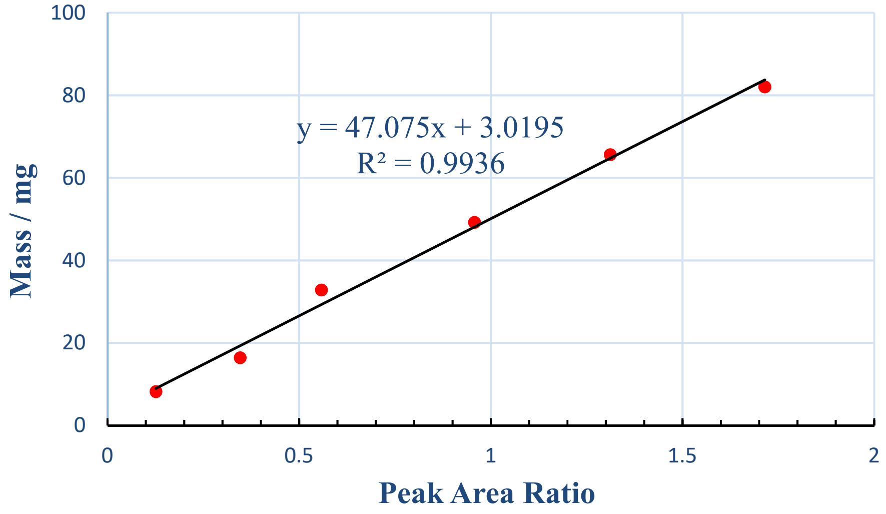

Figure S20 calibration curve of bis(trimethylsilyl)carbodiimide (BTMSC) content. 10.0-100.0 mg of BTMSC was added to a solution of dodecane (40 µL)/ethyl acetate in a total volume of 4.0 mL.

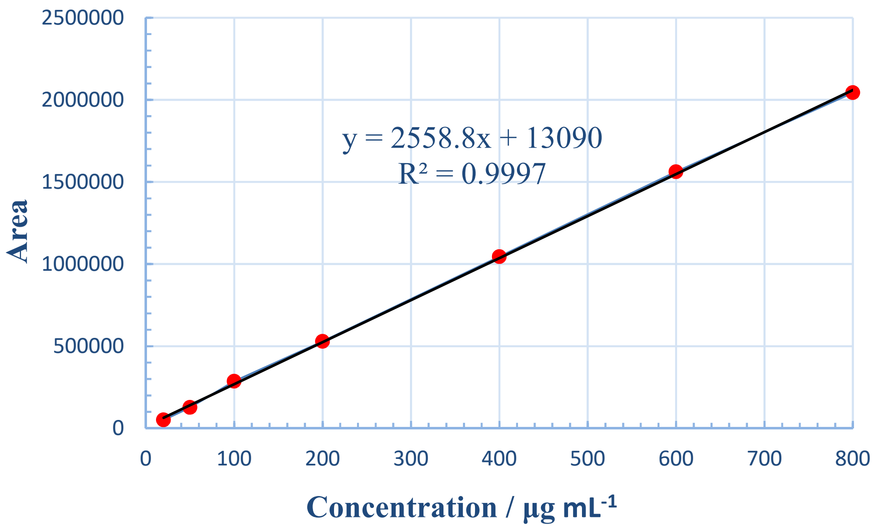

Figure S21 relationship between the concentration of urea and the peak area of UV absorption at 195 nm. The linear regression equation was y = 2558.8x+13090 (R^2 = 0.9997), where x is the concentration of urea, y is the peak area of UV absorption at 195 nm.

Table S1 the effects of different solvents for synthesizing BTMSC at room temperature

Time	GC Yield					
	Toluene	Hexane	Et_2O	Acetone	Acetonitrile	THF
1 h	8 %	16 %	11 %	43 %	71 %	72 %
2 h	28 %	26 %	45 %	33 %	69 %	61 %
3 h	25 %	44 %	59 %	18 %	52 %	58 %

Table S2 the yield of BTMSC in mixed Et_2O/Acetone solution (3.5 mL/0.5 mL) at room temperature

Time	Yield
0.5 h	45 %
1 h	63 %
1.5 h	84 %
2 h	78 %
2.5 h	74 %
3 h	72 %

Table S3 the stability of Li_2CN_2 in different conditions at room temperature

	H_2O [a]	EtOH [b]	MeOH [c]	Air [d]
Yield	12 %	23%	29 %	63 %

[a] Li_2CN_2 was stirred in water for 1 h; [b] Li_2CN_2 was stirred in ethanol for 1 h; [c] Li_2CN_2 was stirred in methanol for 1 h; [d] Li_2CN_2 was exposed to air for 7 h. Before being treated with TMSCl in mixed Et_2O/acetone solution (3.5 mL/0.5 mL), the solution was dried *in vacuo* to remove any remaining protonic solvents.

Table S4 the yield of urea at different time points at room temperature

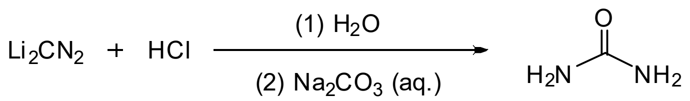

Time	Yield		
	3.0 equiv. HCl	5.0 equiv. HCl	7.0 equiv. HCl
1 h	1 %	6 %	9 %
2 h	3%	11 %	15 %
4 h	6 %	21 %	28 %
21 h	26 %	64 %	75 %

Table S5 the yield of urea at 50 °C

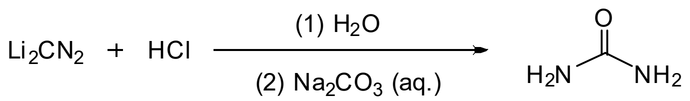

Time	Yield		
	3.0 equiv. HCl	5.0 equiv. HCl	7.0 equiv. HCl
1 h	30 %	72 %	78 %
2 h	48 %	91 %	92 %
3 h	63 %	98 %	98 %
4 h	73 %	101 %	99 %
5 h	83 %	109 %	96 %
6 h	87 %	95 %	89 %

Reference

51. Yang, J.-M. et al. Insertion of alkylidene carbenes into B–H bonds. *J. Am. Chem. Soc.* **142**, 20924–20929 (2020).
